# Plasma cell-free RNA profiling distinguishes cancers from pre-malignant conditions in solid and hematologic malignancies

**DOI:** 10.1038/s41698-022-00270-y

**Published:** 2022-04-25

**Authors:** Breeshey Roskams-Hieter, Hyun Ji Kim, Pavana Anur, Josiah T. Wagner, Rowan Callahan, Elias Spiliotopoulos, Charles Ward Kirschbaum, Fehmi Civitci, Paul T. Spellman, Reid F. Thompson, Khashayar Farsad, Willscott E. Naugler, Thuy T. M. Ngo

**Affiliations:** 1 Cancer Early Detection Advanced Research Center (CEDAR), Knight Cancer Institute, Oregon Health and Science University, Portland, OR US; 2 Department of Biomedical Engineering, Oregon Health and Science University, Portland, OR US; 3 Department of Molecular and Medical Genetics, Oregon Health and Science University, Portland, OR US; 4 Computational Biology Program, Oregon Health and Science University, Portland, OR US; 5 Department of Radiation Medicine, Oregon Health and Science University, Portland, OR US; 6 Division of Hospital and Specialty Medicine, VA Portland Healthcare System, Portland, OR US; 7 Dotter Department of Interventional Radiology, Oregon Health and Science University, Portland, OR US; 8 Division of Gastroenterology and Hepatology, School of Medicine, Oregon Health and Science University, Portland, OR US

**Keywords:** Diagnostic markers, Cancer genomics, Hepatocellular carcinoma, Myeloma

## Abstract

Cell-free RNA (cfRNA) in plasma reflects phenotypic alterations of both localized sites of cancer and the systemic host response. Here we report that cfRNA sequencing enables the discovery of messenger RNA (mRNA) biomarkers in plasma with the tissue of origin-specific to cancer types and precancerous conditions in both solid and hematologic malignancies. To explore the diagnostic potential of total cfRNA from blood, we sequenced plasma samples of eight hepatocellular carcinoma (HCC) and ten multiple myeloma (MM) patients, 12 patients of their respective precancerous conditions, and 20 non-cancer (NC) donors. We identified distinct gene sets and built classification models using Random Forest and linear discriminant analysis algorithms that could distinguish cancer patients from premalignant conditions and NC individuals with high accuracy. Plasma cfRNA biomarkers of HCC are liver-specific genes and biomarkers of MM are highly expressed in the bone marrow compared to other tissues and are related to cell cycle processes. The cfRNA level of these biomarkers displayed a gradual transition from noncancerous states through precancerous conditions and cancer. Sequencing data were cross-validated by quantitative reverse transcription PCR and cfRNA biomarkers were validated in an independent sample set (20 HCC, 9 MM, and 10 NC) with AUC greater than 0.86. cfRNA results observed in precancerous conditions require further validation. This work demonstrates a proof of principle for using mRNA transcripts in plasma with a small panel of genes to distinguish between cancers, noncancerous states, and precancerous conditions.

## Introduction

Although recent advances in cancer research offer new methods to treat cancer, early detection of malignancy still confers the highest chance of improving long-term patient survival. Currently, only 2.4% of metastatic liver cancer patients survive for more than 5 years^1^. Early detection of liver cancer, which has the most rapidly increasing incidence in the United States, has the potential to extend 5-year survival rates to 33% with current treatment options. Even with hematologic malignancies like multiple myeloma (MM), 95% of patients are diagnosed when cancer has already spread systemically, resulting in at least a 20% decrease in 5-year survival rates compared to detection at earlier stages^2^. Noninvasive, low cost and reliable cancer diagnostic assays could greatly benefit patients by facilitating accessibility to early cancer screening.

In many cancers, there are disease states known to be precursors of malignant disease. For example, MM, a cancer of antibody-producing plasma cells, is often preceded by monoclonal gammopathy of undetermined significance (MGUS), which is characterized by lower levels of abnormal antibodies. The prevalence of MGUS is about 3% in the Caucasian population, and the conversion rate from MGUS to multiple myeloma is ~1% per year^3,4^. Hepatocellular carcinoma (HCC), the most common form of liver cancer, is often preceded by liver cirrhosis (Cirr) characterized by irreversible fibrosis of the liver. The prevalence of cirrhosis is between 4.5–9.5% of the global population^5–7^. The risk of developing *de novo* HCC in patients with liver cirrhosis ranges between 1 and 5% per year, depending on the etiology of the cirrhosis^5–11^. Most early cancer detection studies to date have focused on distinguishing cancer from healthy controls, rather than discriminating between cancer and common premalignant conditions. Therefore, there is an unmet clinical need for a simple blood test that can identify patients with premalignant conditions who require further intervention due to a higher likelihood of cancer incidence.

With current clinical practices, a cancer diagnosis is primarily initiated based upon costly imaging studies or invasive screening procedures. Alternatively, some cancers may only come to attention with clinical symptoms that present at more advanced stages. Liquid biopsy, a minimally invasive method for sampling and analyzing biomarkers in various body fluids, has the potential to improve cancer diagnosis and prognosis^12–15^. Several blood-based analytes have been explored for use in liquid biopsies for cancer detection such as circulating cells (circulating tumor cells (CTCs), circulating hybrid cells (CHCs), tumor-associated macrophages (TAMs))^16–21^, circulating tumor DNA (ctDNA)^22–24^, platelets^25–27^, and protein panels^28^. However, ctDNA and circulating cells are present at low levels, have varied characteristics between patients, and only weakly correlate with phenotypic changes in cancer^17,29,30^. Epigenetic features of ctDNA such as methylation and 5-hydroxymethylcytosine signatures, or ctDNA protected patterns may provide information about the tissue of origin for pan-cancer detection^31–38^. However, these methods may require large deep sequencing coverage to be effective and may have inadequate sensitivity and specificity. Recent transcriptome analysis of tumor-educated platelets has shown promise for pan-cancer detection^25–27^, but platelets are fragile, can be easily activated in vitro, and have highly variable characteristics depending on their preparation which make them challenging to utilize with existing clinical blood tests^39^. There is thus a need for robust liquid biopsy technology that can overcome these challenges in a safe, reliable, and cost-effective manner.

Circulating cell-free RNA (cfRNA) in the blood is released from cells by active secretion or through apoptosis and necrosis^40,41^. Plasma cfRNA has the potential to reflect the systemic response to growing tumors and provide information about the tissue of tumor origin specifically by cancer type. Previous work has demonstrated that global cfRNA profiles indicate temporal changes in organ-specific transcripts. Further analysis of these transcripts facilitated the prediction of pregnancy delivery, preterm birth, and distinction of cancer from healthy controls^42–46^. Here, we explore the potential of cfRNA profiles to distinguish cancers from their premalignant conditions. We sequenced total plasma cfRNA from plasma samples of patients with HCC and MM and their precancerous conditions including liver cirrhosis (Cirr) and MGUS, and non-cancer (NC) donors. We identified potential cfRNA biomarkers using plasma cfRNA-sequencing of a pilot sample set and validated potential cfRNA biomarkers in an independent sample set. We further validated the sequencing data using orthogonal measurement by quantitative reverse transcription PCR. Feature selection and classification models were built to explore the potential of cfRNA profiles in differentiating malignant from premalignant conditions.

## Results

### Identification of plasma cfRNA biomarkers by sequencing

To identify cfRNA transcripts which potentially distinguish cancer patients from NC individuals, we prospectively collected blood samples from the following individuals: a pilot set of ten MM and eight HCC patients; 13 patients with premalignant conditions including eight MGUS and four Cirr; and 20 NC donors. Detailed clinical information of the samples is listed in the supplementary information (Supplementary Table 1). Plasma cfRNA samples were sequenced to saturation with a mean of 33.8 M raw reads with a range of 27.7 to 52.3 M (Supplementary Table 2 and Supplementary Figs. 2, 3a). After selecting for reads that mapped uniquely to the human genome, the cfRNA libraries had an average read depth of 14 M with a range from 2.3 to 43 M. On average, 80% of reads mapped to exons (Supplementary Table 2 and Supplementary Fig. 3b). A total of 39,374 annotated features were detected with at least one mapped read across all samples. The majority of detected RNAs were protein-coding with a mean fraction of 82% and a range from 65 to 89% (Supplementary Table 2 and Supplementary Fig. 3c). The fraction of reads mapping to exons and the distribution of read depths were uniform across all sample groups (Fig. S3b, c).

**Fig. 1:**
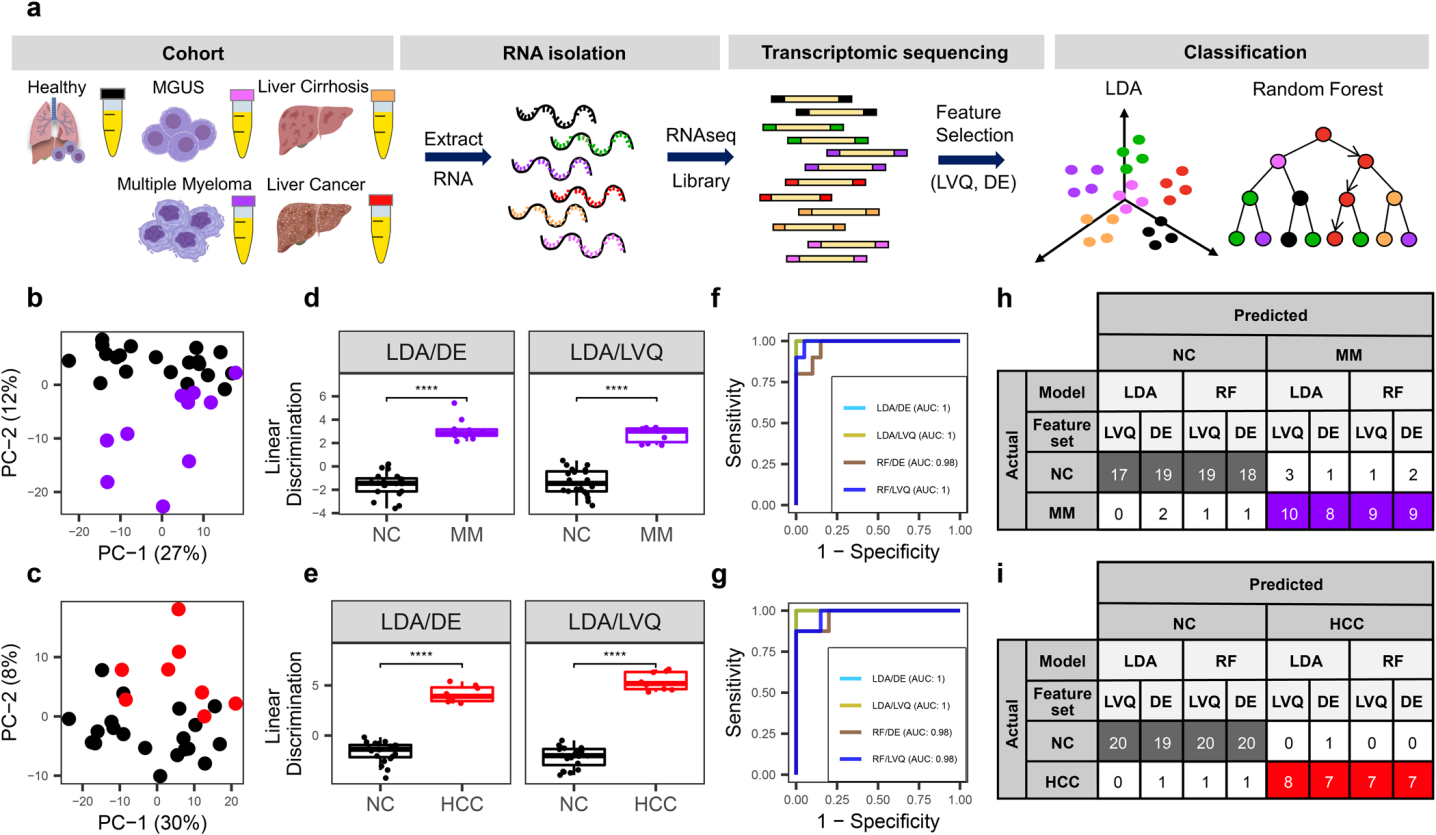
cfRNA profiles distinguish between cancer vs. healthy donors. **a** Schematic overview of the cfRNA profiling workflow starting from plasma collected from the patients and NC donors in EDTA-coated tubes, cfRNA extraction, sequencing, feature selection, and classification. **b**, **c** PCA analysis using the top 500 genes with the largest variance across NC and MM (**b**) or HCC samples (**c**). **d**, **e** Linear discriminant analysis (LDA) using DE genes with *p*_adj_ <0.01 and top ten most important genes identified by LVQ analysis. *P* value is derived from the Wilcoxon test. Center-line indicates the median value across all patients in that group, and the hinges represent the lower (Q1) and upper (Q3) quartile, with whiskers extending to the minimum and maximum of the resulting distribution. **f**, **g** ROC curves of the two classification models LDA and Random Forest (RF) model with two feature sets DE and LVQ. **h**, **i** LOOCV with the two models LDA and RF with two feature set DE and LVQ. DE genes are listed in Supplementary Table 3 and LVQ genes are listed in Supplementary Table 5.

We then determined if cfRNA profiles can distinguish HCC and MM from NC donors. Principal component analysis (PCA) using the top 500 genes with the largest variance across all samples through pairwise comparison showed separation of HCC and MM cfRNA profiles from that of NC donors (Fig. 1b, c). Differential expression (DE) analysis of the pairwise comparison between individual cancer types with respect to NC donors using DEseq2 yielded 110, and 12 differentiating genes (adjusted *p* value <0.01) for MM and HCC, respectively (Supplementary Table 3 and Supplementary Fig. 4a, b). Permutations of random sample shuffling in each pair with 500 rounds resulted in zero significant differentiating genes (*p*_adj_ < 0.01) in more than 95 and 94% of permutations for each pair comparing MM, and HCC to non-cancer donors, respectively (Supplementary Table 4 and Supplementary Fig. 4c, d). Gene ontology analysis revealed that MM upregulated genes were enriched for oxygen transport and gas transport (Supplementary Figure 5a). In HCC, the upregulated gene set was enriched for plasminogen activation (Supplementary Fig. 5b). This data collectively indicates the separation of cfRNA profiles in HCC and MM compared to NC donors.

To further explore the potential of cell-free RNA for cancer detection, we applied linear discriminant analysis (LDA) and a Random Forest (RF) algorithm to find combinations of discriminating genes to separate cancer from non-cancer individuals. Two independent methods were used to identify specific input gene lists for the classifying algorithms. First, discriminating genes using DESeq2 analysis with adjusted *p* value < 0.01 (Supplementary Table 3) were used as one feature set (DE gene set). Second, we implemented the learning vector quantization (LVQ) method to find the most important features that distinguished the two groups and selected the top ten as another feature set (LVQ gene set) (Supplementary Table 5). The linear combination for each gene set by LDA showed significant separation between HCC and MM from NC donors with *p* value of 6.7 × 10^−8^, 6.7 × 10^−10^, and 6.4 × 10^−7^, 6.4 × 10^−7^ using the DE and top ten LVQ gene sets, respectively (Fig. 1d, e). We further employed the Random Forest (RF) method to develop orthogonal classification models. The area under the receiver operating characteristic (ROC) curve (AUC) was higher than 0.92 in both LDA and RF models with both DE and LVQ feature sets for the two cancer types (Fig. 1f, g).

To evaluate the significance and accuracy of our classification models, we employed the leave-one-out cross-validation (LOOCV) method. Both LDA and RF algorithms were trained on the described DE and LVQ gene sets, resulting in four classification models (Fig. 1f–i). Classifying MM from non-cancer donors yielded 90% accuracy (27/30) for all four models tested. HCC was correctly differentiated from NC donors with accuracies of 100% (28/28) and 93% (26/28) using the LDA method or 96% (27/28) and 96% (27/28) using the RF method with LVQ and DE feature sets, respectively. Overall, the LOOCV test confirmed that the biomarker sets determined by DESeq2 and LVQ methods, combined with our classification models using LDA and RF algorithms are statistically significant. LVQ gene sets yielded higher accuracy for both cancer types and were used as the feature sets for further validation.

### cfRNA profiles distinguish multiple myeloma from its premalignant condition, MGUS, and MGUS from non-cancer

**Fig. 2:**
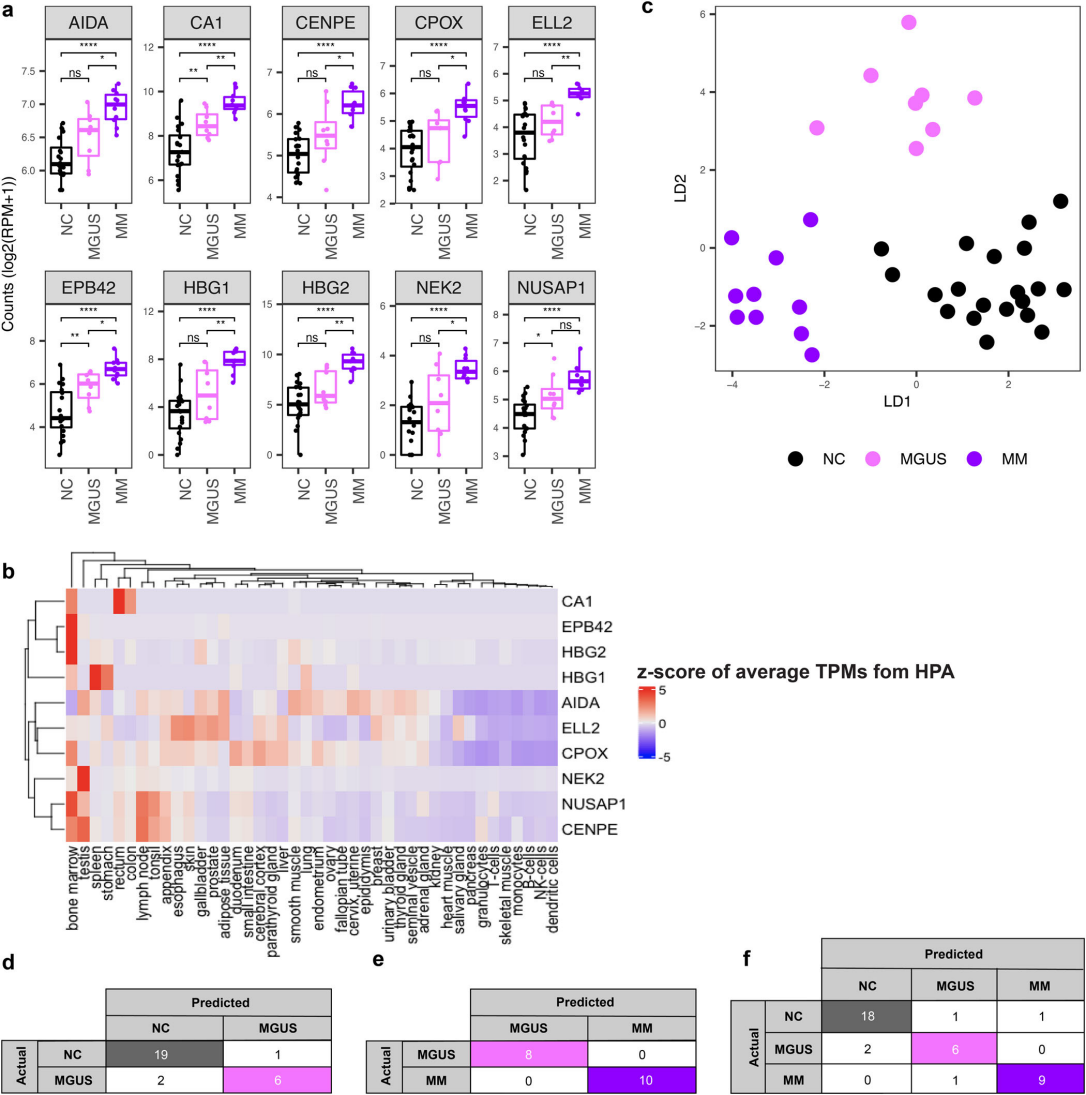
cfRNA profiles distinguish between non-cancer, MGUS, and multiple myeloma donors. **a** Boxplots of representative top ten most significant genes resulted from the LVQ analysis for MM versus NC. *P* value was calculated for each pair by the *t*-test. Center-line indicates the median value across all patients in that group, and the hinges represent the lower (Q1) and upper (Q3) quartile, with whiskers extending to the minimum and maximum of the resulting distribution. **b** Heatmap of *z*-score across publicly available tissue-level expression data from the Human Protein Atlas (HPA) for the top ten LVQ genes identified in MM vs. NC. **c** LDA plot using ten genes from pairwise analysis across NC - MGUS and NC - MM pairs using the LVQ method. **d**–**f** LOOCV using the Random Forest (RF) model with top ten LVQ genes to discriminate MGUS and NC (**d**), MM vs MGUS (**e**), and three groups NC, MGUS, and MM (**f**). Genes included in the RF analysis are listed in Supplementary Table 5.

We next examined if cfRNA profiles were able to recapitulate the transition from a precancerous condition to a cancerous one and distinguish between them. We chose to test our hypothesis on MM as it has the well-defined precancerous condition of MGUS. The top ten most significant genes that discriminate MM from NC donors as identified by LVQ displayed a gradual transition in cfRNA level from the non-cancer donors through MGUS to MM (Fig. 2a). Among these ten most significant genes, seven genes (CA1, EPB42, HBG1, HBG2, CENPE, CPOX, and NUSAP1) have higher expression in bone marrow, where cancerous plasma cells accumulate, compared to other tissue and cell types in publicly available data from the Human Protein Atlas^47,48^ (Fig. 2b). Three genes resulting from the LVQ analysis are related to cell cycle processes: Centromere protein E (CENPE), a kinesin-like motor protein that accumulates in the G2 phase of the cell cycle and is highly expressed in the bone marrow^49,50^; Serine/threonine-protein kinase (NEK2), which is involved in mitotic regulation^50,51^; and Nucleolar and spindle associated protein 1 (NUSAP1), a nucleolar-spindle-associated protein that plays a role in spindle microtubule organization^52^.

An LDA plot using a combination of the top ten LVQ genes from pairwise comparisons MM—NC, and MGUS—NC displayed the separation of all three groups (Fig. 2c). An RF model using the top ten most important LVQ genes from MGUS—NC pairwise comparison yielded an accuracy of 89.3% (19/20 NC donors and 6/8 MGUS patients) (Fig. 2d). Classification of MM from MGUS yielded an accuracy of 100% (8/8 MGUS and 10/10 MM) using LOOCV with the RF classification method using the top ten most important genes from LVQ analysis of MM versus NC comparison as a feature set (Fig. 2e). The three-group classification resulted in an accuracy of 86.8% (18/20 NC, 6/8 MGUS, and 9/10 MM) defined by LOOCV using the RF method with the feature set composed of the combination of the top 10 LVQ genes from the comparison MM versus NC and MGUS versus NC donors (Fig. 2f).

### cfRNA profiles distinguish liver cancer from its premalignant condition, cirrhosis, and cirrhosis from non-cancer

**Fig. 3:**
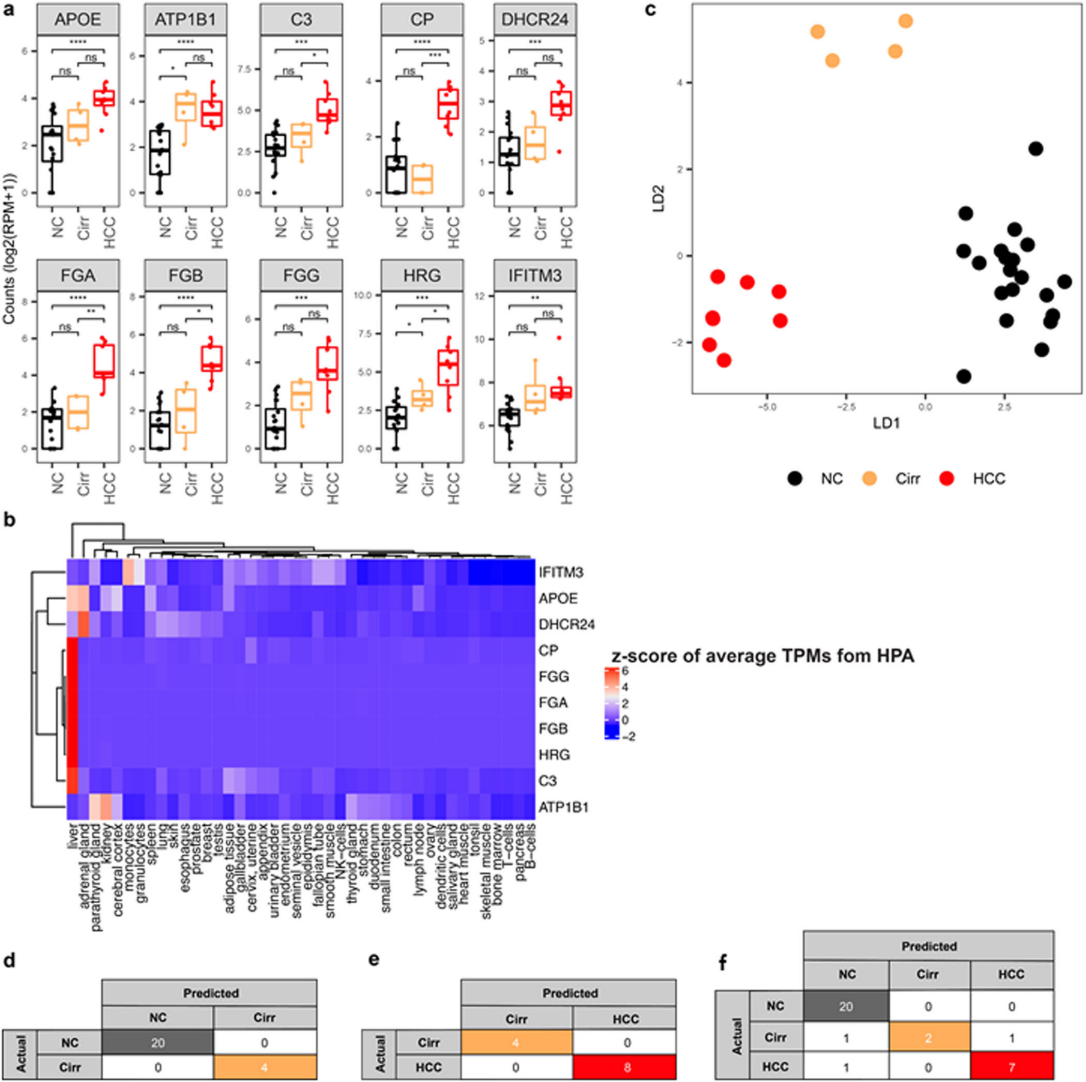
cfRNA profiles distinguish between non-cancer, liver cirrhosis, and liver cancer donors. **a** Boxplots of representative top ten most significant genes resulted from the LVQ analysis for HCC vs. NC. *P* value was calculated for each pair by the *t*-test. Center-line indicates the median value across all patients in that group, and the hinges represent the lower (Q1) and upper (Q3) quartile, with whiskers extending to the minimum and maximum of the resulting distribution. **b** Heatmap of *z*-score across publicly available tissue-level expression data from the Human Protein Atlas (HPA) for the top ten LVQ genes identified in HCC vs. NC (**c**) LDA plot using top ten genes identified from each pairwise analysis between NC - Cirr and NC - HCC samples using the LVQ method. **d**–**f** LOOCV using the RF model with top ten LVQ genes to discriminate Cirr and NC (**d**), HCC vs Cirr (**e**), and three groups NC, Cirr, and HCC (**f**). Genes included in the RF analysis are listed in Supplementary Table 5.

Next, we asked if we could distinguish between a solid tumor such as HCC and its precancerous condition, Cirr. Among the top ten most important genes that discriminate HCC from NC identified by the LVQ analysis, five genes also significantly differentiate HCC from Cirr (Fig. 3a). Interestingly, eight out of the top ten genes are expressed specifically in the liver and the corresponding proteins are secreted into the blood^47,48^ (Fig. 3b). Apolipoprotein E (APOE) binds to the specific liver and peripheral cell receptors and is essential for the normal catabolism of triglyceride-rich lipoprotein constituents^53^. Complement C3 (C3) is synthesized in the liver and secreted to the plasma and is involved in both innate and adaptive immune responses^54^. Ceruloplasmin (CP) is a secreted plasma metalloprotein from the liver that binds copper in the plasma and is involved in the peroxidation of Fe(II) transferrin to Fe(III) transferrin^55^. 24-dehydrocholesterol reductase DHCR24 catalyzes the reduction of sterol intermediates^56^. Fibrinogen alpha chain (FGA), fibrinogen beta chain (FGB), and fibrinogen gamma chain (FGG) encodes the coagulation factor fibrinogen, which is a component of blood clotting^57^. Histidine-rich glycoprotein (HRG) is a plasma glycoprotein that binds heparin sulfate on the surface of the liver, lung, kidney, and heart endothelial cells^58^.

We explored the potential of cfRNA to distinguish HCC from Cirr and Cirr from NC individuals. An LDA plot using the feature set comprised of a combination of the top 10 LVQ genes identified for the pairwise comparisons of HCC—NC and Cirr—NC, shows a distinct separation between these groups (Fig. 3c). RF methods using the top ten important genes from Cirr—NC pairwise comparisons yielded 100% accuracy in classifying Cirr from NC samples using LOOCV (Fig. 3d). Classification of HCC from Cirr also yielded 100% accuracy using LOOCV with RF (Fig. 3e). We further attempted to classify three classes including NC, Cirr, and HCC in one model. The three-group classification resulted in 90.6% accuracy using LOOCV with RF (Fig. 3f).

### Validation of cfRNA biomarkers

**Fig. 4:**
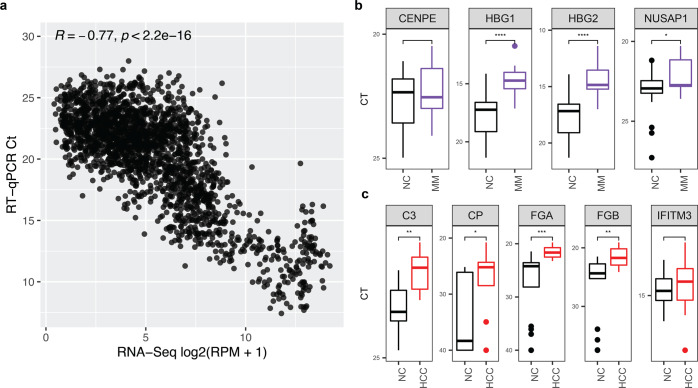
qRT-PCR of cfRNA biomarkers is concordant with RNA-sequencing data. **a** Correlation plot of qRT-PCR data compared to RNA-sequencing data. *P* value was calculated by t-test. **b**, **c** qRT-PCR Ct values of top four LVQ genes identified from MM versus NC (**b**) and top 5 LVQ genes identified from HCC versus NC (**c**). Center-line for boxplots in both **b** and **c** indicates the median value across all patients in that group, and the hinges represent the lower (Q1) and upper (Q3) quartile, with whiskers extending to the minimum and maximum of the resulting distribution.

We designed a primer panel for the LVQ gene set to validate the sequencing data by quantitative reverse transcription PCR (RT-qPCR). RT-qPCR results from the pilot sample set were consistent with the sequencing data with a Pearson correlation coefficient >0.77 and a *p* value of 2.2 × 10^−16^ (Fig. 4a). We confirmed that the differential levels of cfRNA transcripts of genes identified by the LVQ algorithm (HBG1, HBG2, NUSAP1, for MM and C3, CP, FGA, FGB for HCC) from RNA-sequencing were also observed with RT-qPCR (Fig. 4b).

**Fig. 5:**
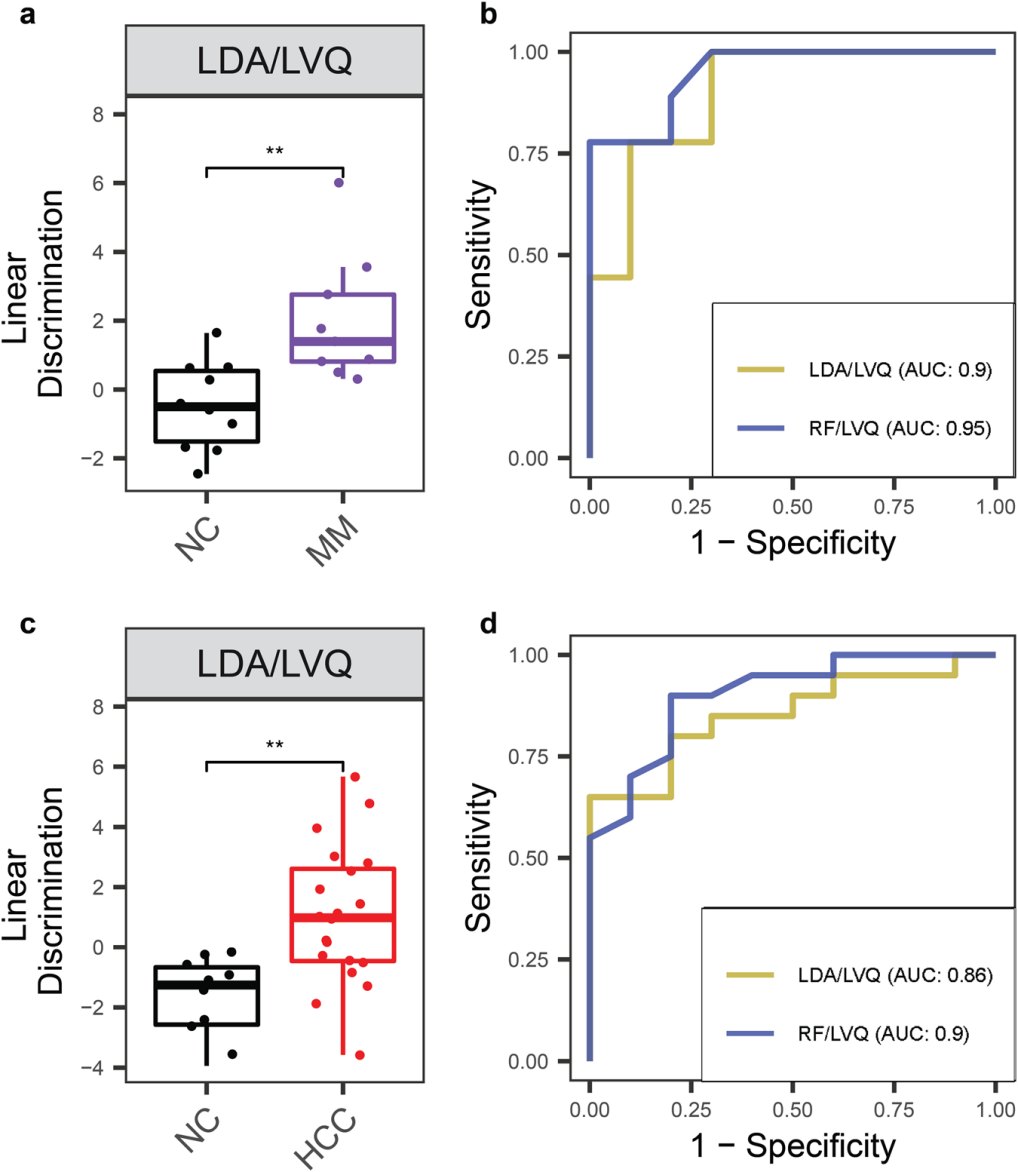
cfRNA biomarkers and classification models validated in independent sample set. **a**, **c** Linear discriminant analysis in the validation cohort using top ten LVQ genes identified and classification models trained on the pilot cohort for MM versus NC, and HCC versus NC. *P* value was calculated for each pair by the Wilcoxon rank-sum test. Center-line indicates the median value across all patients in that group, and the hinges represent the lower (Q1) and upper (Q3) quartile, with whiskers extending to the minimum and maximum of the resulting distribution. **b**, **d** ROC curves of these same classification models, trained on the pilot sample set and tested with the validation sample set, using the top ten LVQ genes identified from the pilot sample set.

To confirm that the feature sets and classification models defined in our pilot cohort were robust and generalizable, we collected a set of independent validation samples from ten NC controls, nine MM patients, and 20 HCC patients (Supplementary Table 1 and Supplementary Figs. 6, 7). We validated the cfRNA biomarkers identified from the pilot set in silico by measuring the classification accuracy on this independent sample set using the models trained with the pilot dataset using the LVQ gene sets. The linear combination identified by LDA in the pilot cohort of the LVQ feature set showed significant separation in the validation sample set between MM and HCC from NC donors, consistent with our previous results (Fig. 5a, c). Furthermore, both LDA and RF models trained on the pilot cohort with this same feature set were able to classify cancer from NC controls in our validation cohort, with an AUC >0.86 and 0.9 when classifying NC donors from MM and HCC, respectively (Fig. 5b, d).

**Fig. 6:**
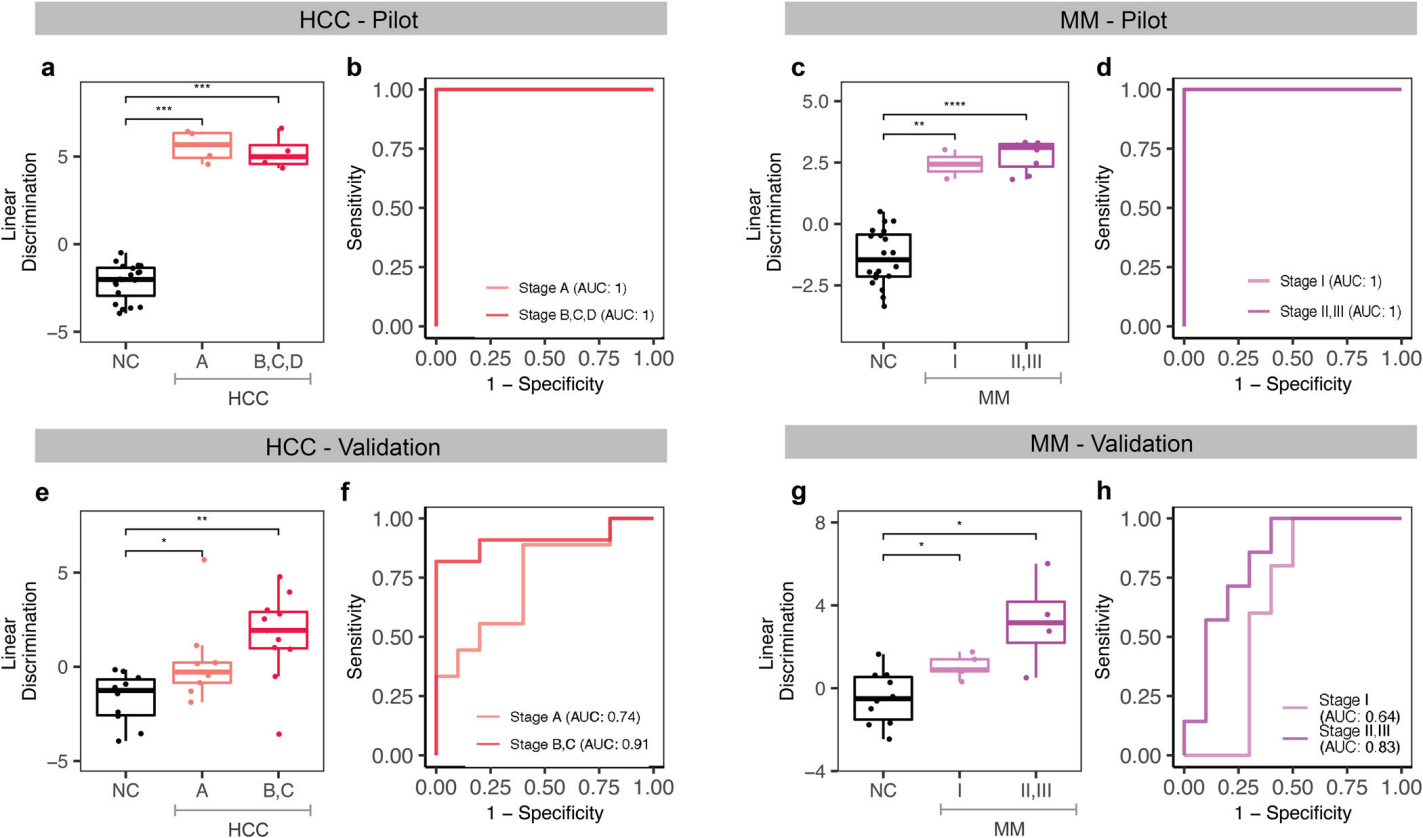
cfRNA biomarkers show clinical stage-dependent discrimination in pilot and validation sample sets. **a**–**d** Linear discriminant analysis using the top ten LVQ genes and model trained in the pilot cohort shows significant discrimination and classification by clinical stage in both HCC (**a**, **b**) and MM (**c**, **d**). **e**–**h** When classifying the independent validation cohort with these same models, we see stage-dependent classification for both HCC (**e**, **f**) and MM (**g**, **h**). *P* value for each pair in (**a**, **c**, **e**, **g**) was calculated by the Wilcoxon rank-sum test, and elements of the boxplots include the median value across all patients in that group shown by the center-line and the hinges which represent the lower (Q1) and upper (Q3) quartile, with whiskers extending to the minimum and maximum of the resulting distribution.

**Fig. 7:**
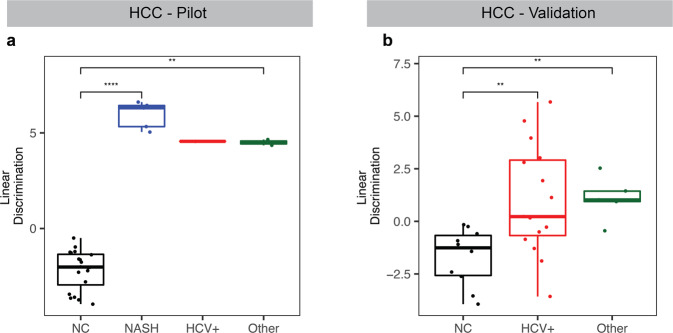
cfRNA biomarkers for HCC show discrimination between various etiologies. **a** Linear discriminant analysis trained on the pilot cohort with the top ten LVQ genes show significant discrimination between NC and HCC on the background of NASH, HCV+, and other etiologies in the pilot cohort and the validation cohort (**b**). *P* value for each pair was calculated by the Wilcoxon rank-sum test. Center-line in each boxplot indicates the median value across all patients in that group, and the hinges represent the lower (Q1) and upper (Q3) quartile, with whiskers extending to the minimum and maximum of the resulting distribution.

Our cfRNA classification model performed well for early and late clinical stages in the pilot set (Fig. 6a–d). In the validation sample set, the model displayed stage-dependent discrimination. It was validated with an AUC of 0.74 for Barcelona Clinic Liver Cancer (BCLC) stage A in HCC (Fig. 6e, f) and an AUC of 0.64 for stage I in MM (Fig. 6g, h). For later stages, the model achieved a higher AUC of 0.91 for BCLC stages B and C in HCC (Fig. 6e, f) and 0.83 for stages II and III in MM (Fig. 6g, h) in the validation sample set. This stepwise increase in discrimination suggests that these biomarkers become more prevalent with cancer progression. HCC classification also showed significant discrimination compared to NC for different etiologies (Fig. 7), and both HCC and MM showed discrimination for males and females (Supplementary Fig. 9) and are not age-dependent (Supplementary Fig. 9) in our pilot and validation sample sets.

## Discussion

We sequenced cfRNA from patients with two cancer types, one solid (HCC) and the other hematologic (MM), and their precancerous conditions (Cirr and MGUS, respectively), and from NC donors. Both cancer types can be distinguished from non-cancer controls and precancerous conditions using their cfRNA profiles. To differentiate each cancer type from individuals without cancer, the combination of ten genes identified by learning vector quantization (LVQ) analysis in each pairwise comparison yielded higher accuracy compared to the use of a larger set of differentiating genes as evaluated by leaving one out cross-validation (LOOCV). RT-qPCR confirmation for a panel of selected biomarkers was consistent with the sequencing data. Plasma cfRNA biomarkers identified from the sequencing data were further validated in an independent sample cohort. The use of a small gene panel potentially enables a cost-effective office-based assay for pan-cancer detection that can be highly useful in broad clinical applications.

To date, most investigations into the potential of blood-based methods for cancer detection have only focused on distinguishing cancers from healthy controls^15,22,25,26,28,36^. However, many cancer types have etiologies associated with precursor states such as MGUS for MM and Cirr for HCC. Here, we report that cfRNA profiles can recapitulate the transition from a precancerous condition to cancer, and can effectively do so for both solid and hematologic cancers. We, therefore, propose that cfRNA panels containing a small number of genes may distinguish cancers from premalignant conditions and precursors from healthy individuals. This development might potentially enable a cost-effective screening strategy for early cancer detection during routine exams in high-risk patients.

Liver and bone marrow have been reported to contribute heavily to the abundance of cell-free nucleic acids in plasma^42,45,46^. This may explain the source of cfRNA biomarkers found in these cancer types. In HCC, eight out of the top ten genes used in the classification model are specifically synthesized in the liver and encode secreted proteins found in the blood that mediate plasminogen activation and fibrinolysis processes. In MM, seven out of ten genes among the most important cfRNA biomarkers have relatively high expression in bone marrow compared to other tissue and cell types and are related to cell cycle processes. These findings indicate that the identified cfRNA biomarkers potentially originate from the tissue of origin of the tumor. Further investigation is needed to better define the tissue and cell-type origin of the biomarkers, and how they may associate with disease initiation and progression.

Our study has important limitations. This is cross-sectional single sampling with a small sample size for both the discovery and validation sets. Furthermore, the sample sets do not represent the wider distribution of cancer subtypes and precursor lesions in the overall population with different underlying etiologies. Another limitation is that the majority of patients and controls are white, so further studies are needed to examine if these results can be extrapolated to a more diverse population of other races. However, accurate classification is not sex- or age-dependent. Although the control population has a higher female/male ratio than the cases, our classification model showed significant discrimination for both males and females in the pilot and validation sets. Despite the median age of controls being 9 and 6 years younger than cases for the pilot and validation sets, respectively, discrimination does not depend on age. Our results for precancerous conditions are promising but require further validation. In addition, we have not fully characterized the stability of cell-free RNA and the biological origin of the identified cfRNA biomarkers. Before the tests developed from this work can be clinically applied, large-scale clinical studies will be required to validate the potential of cfRNA as a cancer biomarker and to build robust classification models. Such large-scale clinical studies will also help to determine if the test can be applied to a broader risk population without specific predispositions.

In summary, we report a proof of principle that global profiling of cell-free mRNA has the potential to establish a platform for longitudinal monitoring of disease progression across both solid and hematologic cancers. This work lays the foundation for developing inexpensive assays that measure transcript levels of mRNA in plasma for a small panel of genes that can differentiate pan-cancer from premalignant conditions and otherwise healthy donors. Intriguingly, organ-specific enriched mRNA transcripts were identified as biomarkers that might indicate the tissue of origin for the tumor. These cell-free plasma RNA biomarkers could be readily combined with other nucleic acid-based and protein-based approaches for potentially increased diagnostic sensitivity and specificity.

## Methods

For a detailed summary of the methods used and the general workflow of our study please see Supplementary Fig. 1.

### Patient samples

Blood samples from non-cancer donors and patients with monoclonal gammopathy of undetermined significance (MGUS), multiple myeloma, liver cirrhosis, and liver cancer were obtained from Oregon Health and Science University (OHSU) by Knight Cancer Institute Biolibrary and Oregon Clinical and Translational Research Institute (OCTRI). All samples were collected under institutional review board (IRB) approved protocols by Oregon Health and Science University. Participants provided written informed consent to take part in the study. Individuals who had no recorded previous history of cancer were considered to be non-cancer donors.

All samples with various diagnoses within the same sample set were collected and processed using a uniform protocol by the same staff at Oregon Health and Science University. The validation set and the pilot set were collected and processed independently by two groups of staff. The clinical information regarding study participants are given in the Supplementaty Table S1. The pilot set includes 10 MM and 8 HCC patients; 13 patients with premalignant conditions including eight MGUS and four Cirr; and 20 NC donors. The validation set includes ten NC controls, nine MM patients, and 20 HCC patients. All Cirr patients underwent abdominal US or MRI and all MGUS patients had an evaluation of bone marrow or assessment of serum-free light chain ratio within 6 months of blood collection for this study.

### Processing of whole blood

For all cohorts, whole blood samples were collected in EDTA-anticoagulated vacutainers. Within 2 h of collection, blood samples were first centrifuged at 1000×*g* for 10 min at 4°C followed by 15,000×*g* for 10 min at 4 °C. Plasma was then stored at −80°C until RNA isolation.

### cfRNA isolation

Samples were randomly shuffled for RNA extraction, library preparation, and sequencing in Illumina flow cells (Fig. 1a). Total RNA purification was performed by using a plasma/serum circulating and exosomal RNA purification kit (Norgen Biotek) from 3 ml of human plasma according to the manufacturer’s protocol. To digest trace amounts of contaminating DNA, RNA was treated with 10X Baseline-ZERO DNase. DNase I treated RNA samples were purified and further concentrated using RNA clean and concentrator-5 (Zymo Research) according to the manufacturer’s manuals. Final eluted RNA was stored immediately at −80 °C.

### Library preparation

We prepared stranded RNA-Seq libraries using Clontech SMARTer stranded total RNA-seq kit v2- pico input mammalian (Takara Bio) according to the manufacturer’s instructions. For cDNA synthesis, we used option 2 (without fragmentation), starting from highly degraded RNA. The input of 7 ul of RNA samples were used to generate cDNA libraries suitable for next-generation sequencing. For the addition of adapters and indexes, we employed SMARTer RNA unique dual index kit −96 U. SMARTer RNA unique dual index of each 5′ and 3′ PCR primer were added to each sample to distinguish pooled libraries from each other. The amplified RNA-seq library was purified by immobilization onto the AMPure XP PCR purification system (Beckman Coulter). The library fragments originated from rRNA and mitochondrial rRNA were treated with ZapR v2 and R-Probes according to the manufacturer’s protocols. For final RNA-seq library amplification, 16 cycles of PCR were performed and the final 20 ul was eluted in Tris buffer following amplified RNA-seq library purification. The amplified RNA-seq library was stored at −20 °C prior to sequencing.

### Sequencing data processing and quality control

Each sample was sequenced to more than 20 million paired-end reads using an Illumina Nextseq or HiSeq sequencer. Adapter sequences were trimmed using sickle tool^59^. After trimming, the quality of the reads were checked using FastQC (v0.11.7)^60,61^ and RSeQC (v2.6.4)^62^. Reads were aligned to the hg38 human genome using the STAR aligner (v2.5.3a)^63^ with two pass mode flag. Duplicated reads were removed using the Picard tool (v1.119)^64^. Read counts for each gene were calculated using the htseq-count tool (v0.11.2)^65^ in intersection-strict mode. The number of mapped reads to each gene were normalized to the total number of reads in the whole transcriptome (Reads Per Million - RPM). For each sample, we calculated exon, intron, intergenic fractions, and protein-coding fractions (CDS exons) using RSeQC^62^ and the read_distribution.py script. Samples with an exon fraction larger than 0.35 were kept for further analysis.

### Identification of cfRNA biomarkers (DESeq and LVQ and GO analysis)

Two independent methods were applied to select cfRNA features for building classification models. Differentiating genes between all pairwise comparisons were identified with the R package DESeq2 (v1.24.0) using the Wald test^66^ with adjusted *p* value (*p*_adj_) < 0.01 (Supplemental Table S3) were used as one feature set (DE gene set). The second method for feature selection uses the LVQ algorithm built-in an R package caret (v6.0-84)—with tenfold cross-validation repeated three times^67^. The top ten most important features were selected by ranking the varImp parameter (LVQ gene set) (Supplemental Table S5). Gene Ontology (GO) analysis was implemented on the top differentiating genes from the DESeq2 analysis with *p*_adj_ < 0.01 using the package topGO (v2.37.0) and a Fischer statistical test to measure significant enrichment of each Gene Ontology term^68^.

### Cancer type classification (LDA and RF)

Two methods were used to build models for classifying cancer types using feature sets identified from pairwise comparison using DESeq2 and LVQ methods. LDA models were built using the R package MASS (v7.3–51.4)^69^. Random Forest models were built using the R package randomForest (v4.6-14)^70^.

### Statistical consideration (permutation test and leave-one-out cross-validation)

To test the significance of the differential expression results for each pairwise comparison of cancer to NC donors, we performed a permutation test in which differential expression analysis between two groups of randomized samples was compared using the DESeq2 package. For each pair, 500 permutations of random shuffling were performed and the number of differentiating genes with *p*_adj_ < 0.01 were documented for building a histogram and compared to the number of significant genes (*p*_adj_ < 0.01) for the group with correct labeling. To determine the significance and accuracy of our classification models, we employed the LOOCV method. Briefly, in LOOCV, LDA, or RF algorithms classified each sample based on the training models obtained from all other samples (total number of samples in each pair minus the testing sample). The test was repeated until all individual samples were classified and cross-validated.

### Tissue specificity of LVQ feature sets using publicly available databases

To evaluate whether our LVQ gene sets were tissue-specific to the tissue of origin (TOO), we downloaded publicly available average tissue-level expression values (transcripts per million; TPMs) from the Human Protein Atlas (ref: https://www.proteinatlas.org/about/download). The methodology used to normalize and calculate average expression values can be found here: https://www.proteinatlas.org/about/assays+annotation#hpa_rna. We then subsetted this matrix of counts values for our two gene sets (top ten LVQ for MM versus non-cancer, and top ten LVQ for HCC versus non-cancer), and calculated a *z*-score across tissue types to evaluate which tissue types the genes were enriched in. Next, we generated a heatmap of this transformed matrix using ComplexHeatmap (v2.4.3).

### Reporting Summary

Further information on research design is available in the Nature Research Reporting Summary linked to this article.

## Supplementary information

Supplemental Material

REPORTING SUMMARY

## Data Availability

cfRNA sequencing data have been deposited in the Gene Expression Omnibus Repository (GSE182824).

## Code availability

In-house scripts used in this manuscript, which includes data processing, downstream analysis, and the scripts used to generate figures is publicly available on Github
