# Supplementary material for: Plasma cell-free RNA profiling distinguishes cancers from pre-malignant conditions in solid and hematologic malignancies
Source: NPJ Precis Oncol. 2022 Apr 25;6:28. doi: 10.1038/s41698-022-00270-y (PMC9038724; doi:10.1038/s41698-022-00270-y)
Supplement: Supplementary file 1 — Supplemental Material [file 41698_2022_270_MOESM1_ESM.pdf]

Plasma cell-free RNA profiling distinguishes cancers from pre-malignant conditions in solid and hematologic malignancies

Breeshey Roskams-Hieter[1,*], Hyun Ji Kim[1,2,*], Pavana Anur[1], Josiah T. Wagner[1], Rowan Callahan[1,2], Elias Spiliotopoulos[1], Charles Ward Kirschbaum[1], Fehmi Civitci[1], Paul Spellman[1,3,4], Reid F. Thompson[1,2,4,5,6], Khashayar Farsad[7], Willscott E. Naugler[7], Thuy T. M. Ngo[1,2,3,✉]

[1]Cancer Early Detection Advanced Research Center (CEDAR), Knight Cancer Institute, Oregon Health and Science University, Portland, OR
[2]Department of Biomedical Engineering, Oregon Health and Science University, Portland, OR
[3]Department of Molecular and Medical Genetics, Oregon Health and Science University, Portland, OR
[4]Computational Biology Program, Oregon Health and Science University, Portland, OR
[5]Department of Radiation Medicine, Oregon Health and Science University, Portland, OR
[6]Division of Hospital and Specialty Medicine, VA Portland Healthcare System, Portland, OR
[7]Dotter Department of Interventional Radiology, Oregon Health and Science University, Portland, OR
[8]Division of Gastroenterology and Hepatology, School of Medicine, Oregon Health and Science University, Portland, OR

[*]Equal contribution

[✉]Corresponding: ngth@ohsu.edu

Supplementary Figures

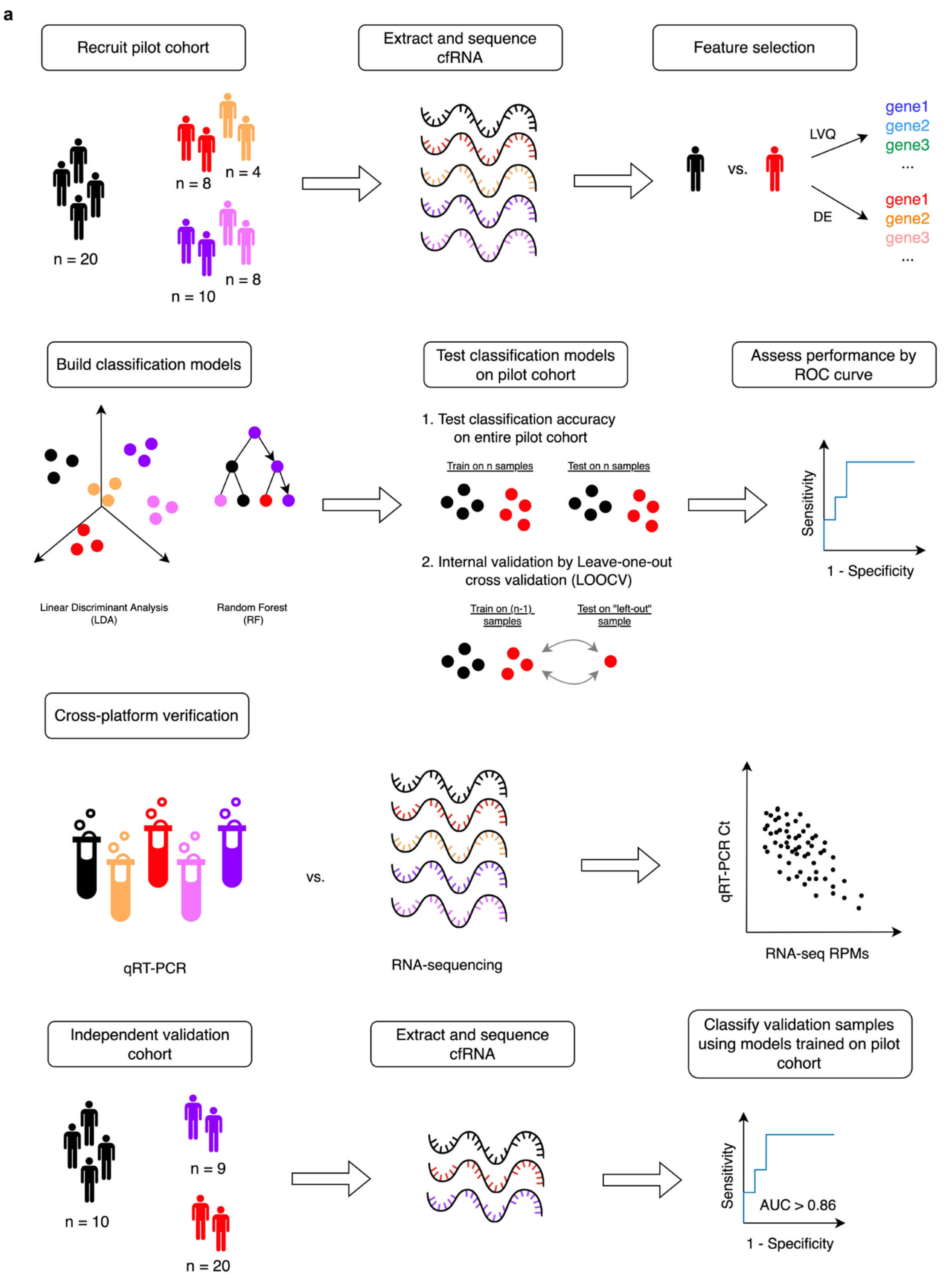

Supplementary Figure 1 | Illustration of overall sample sets and analytic methods used in this study.

a

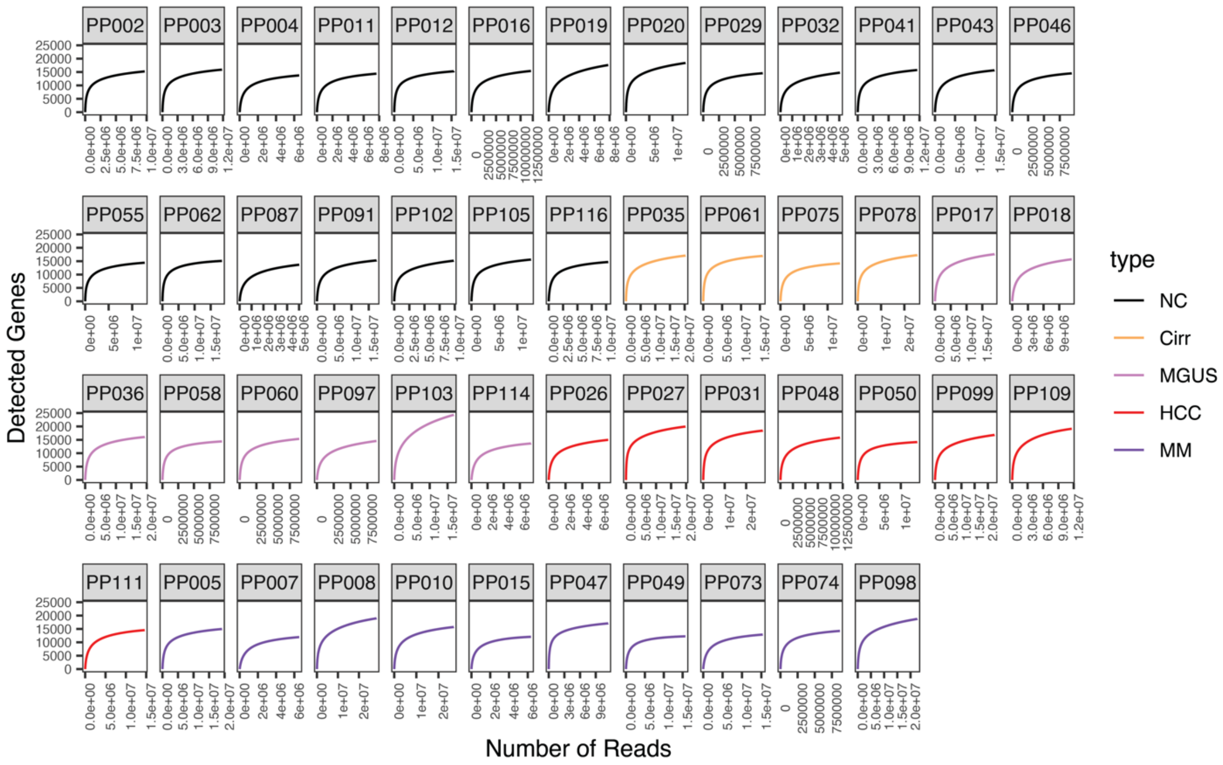

Supplementary Figure 2 | Sequencing saturation analysis of all 50 samples in the pilot cohort. Total reads for each sample were subsampled (x-axis) and the number of genes detected at that read depth is reported (y-axis).

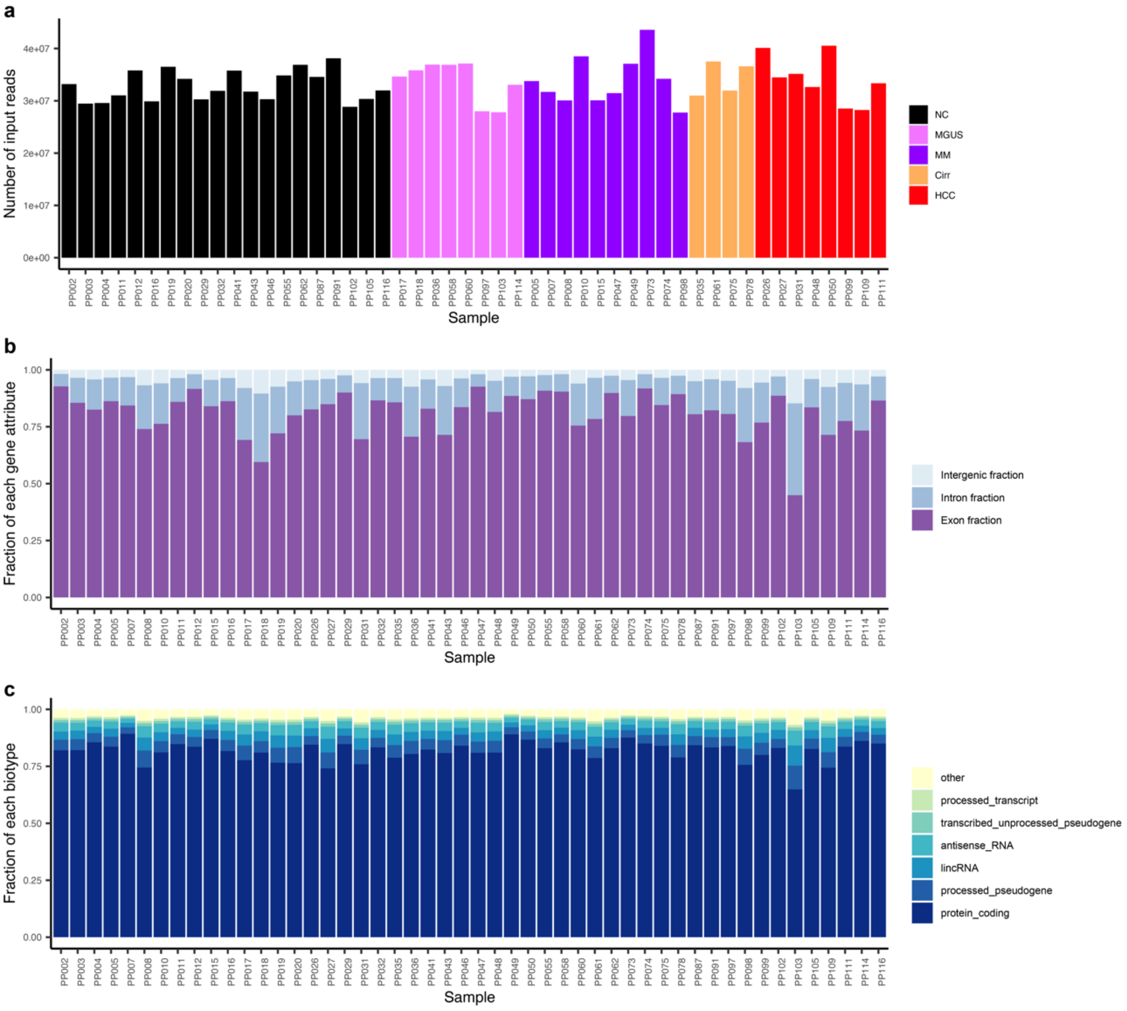

Supplementary Figure 3 | Distribution of (**a**) sequencing reads, (**b**) exon/intron and intergenic fractions, and (**c**) coverage of the transcriptome across all 50 samples in the pilot cohort.

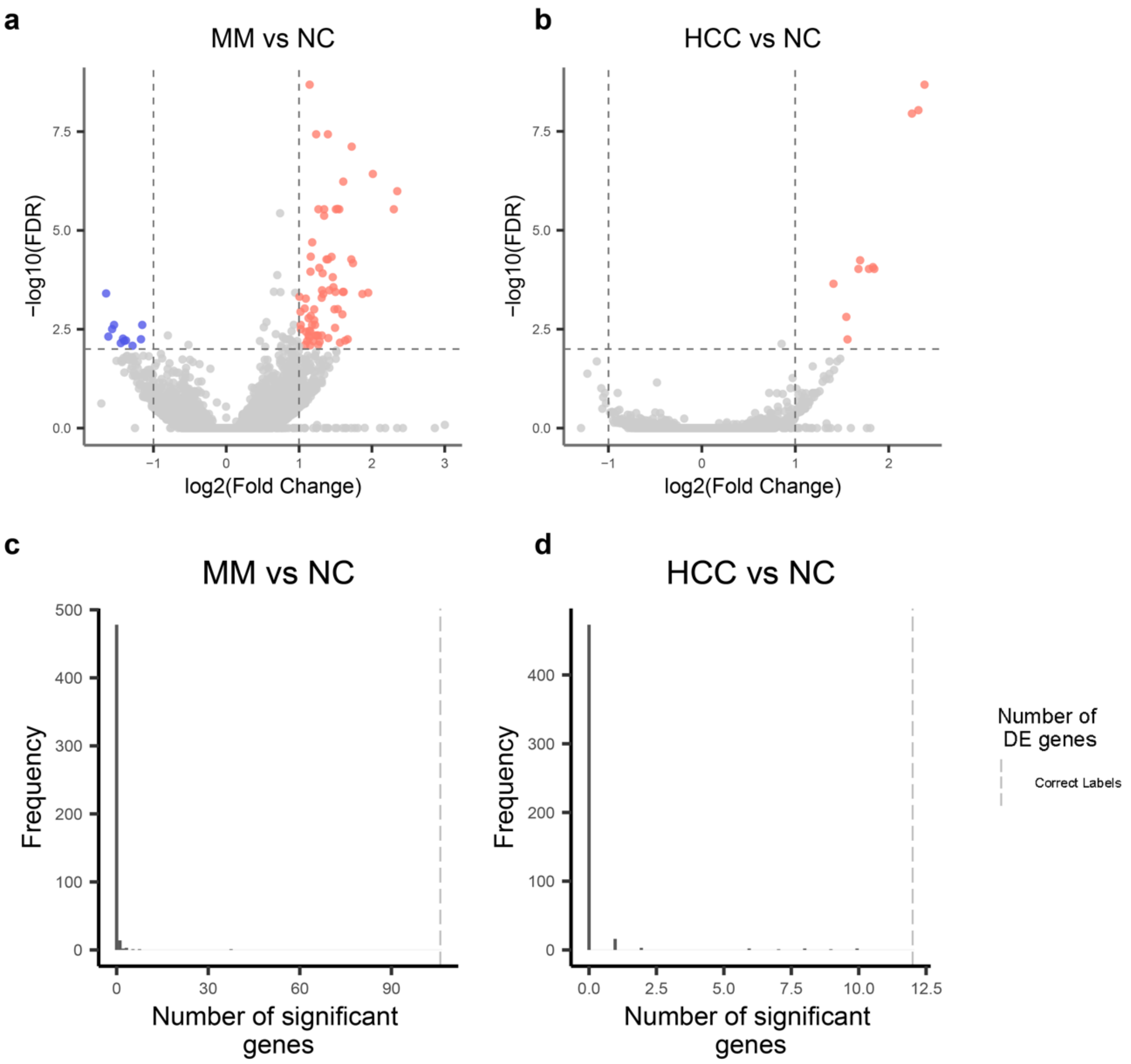

Supplementary Figure 4 | Volcano plots between false discovery rate (FDR) and fold changes for all genes in pairwise comparison between non-cancer (NC) donors and (**a**) multiple myeloma (MM) and (**b**) liver cancer (HCC) analyzed by DESeq2. Histograms of number of significant genes differentiating two groups from random permutation between samples across non-cancer donors and (**c**) multiple myeloma or (c) liver cancer. Differential expression analysis was performed using DESeq2 with Wald test and adjusted p-value cutoff at 0.01. Here we report the results from pairwise comparisons in the pilot cohort.

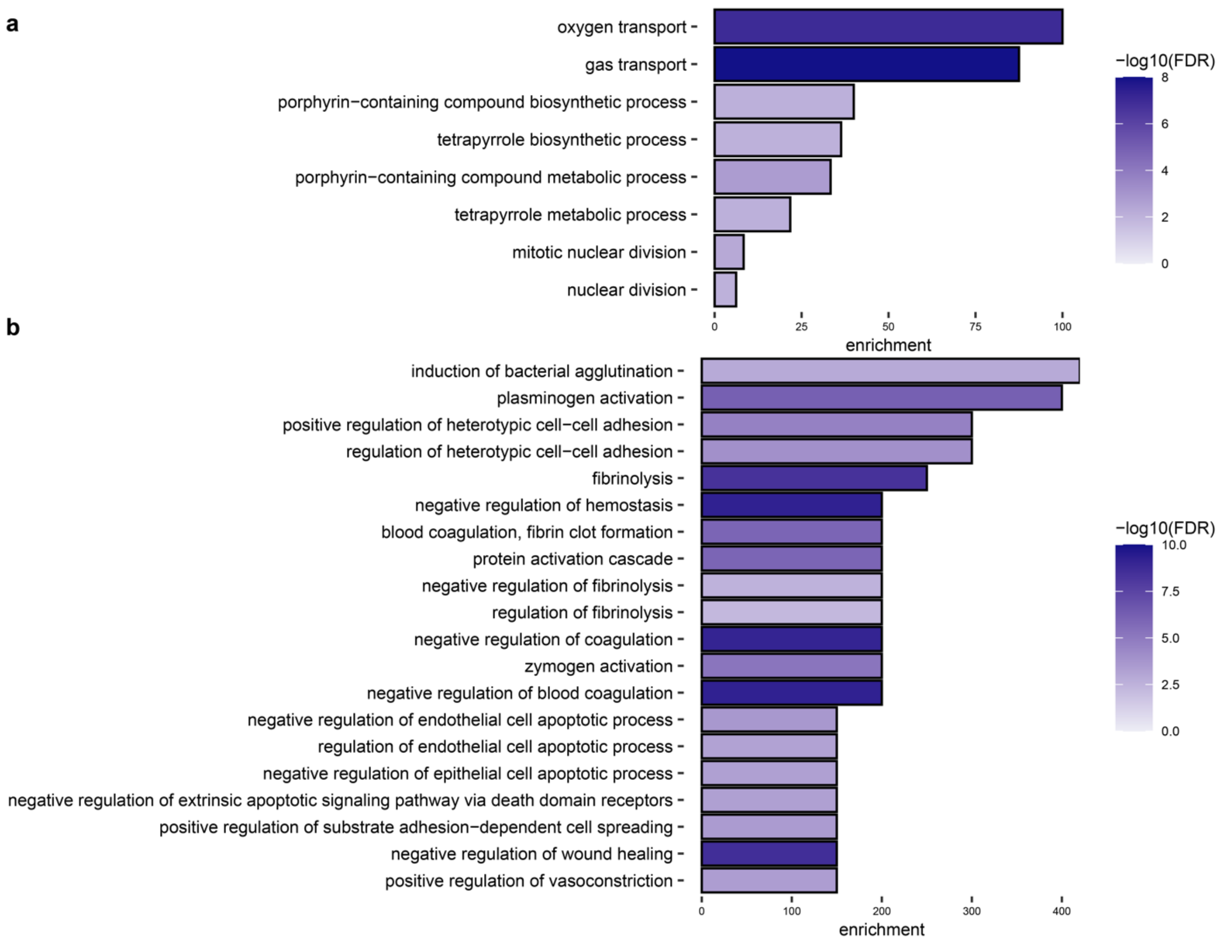

Supplementary Figure 5 | Gene Ontology analysis shows enrichment of biological processes for the significant gene panels identified by DESeq2 analysis for each pairwise comparison between the non-cancer group and (**a**) multiple myeloma and (**b**) liver cancer groups in the pilot cohort.

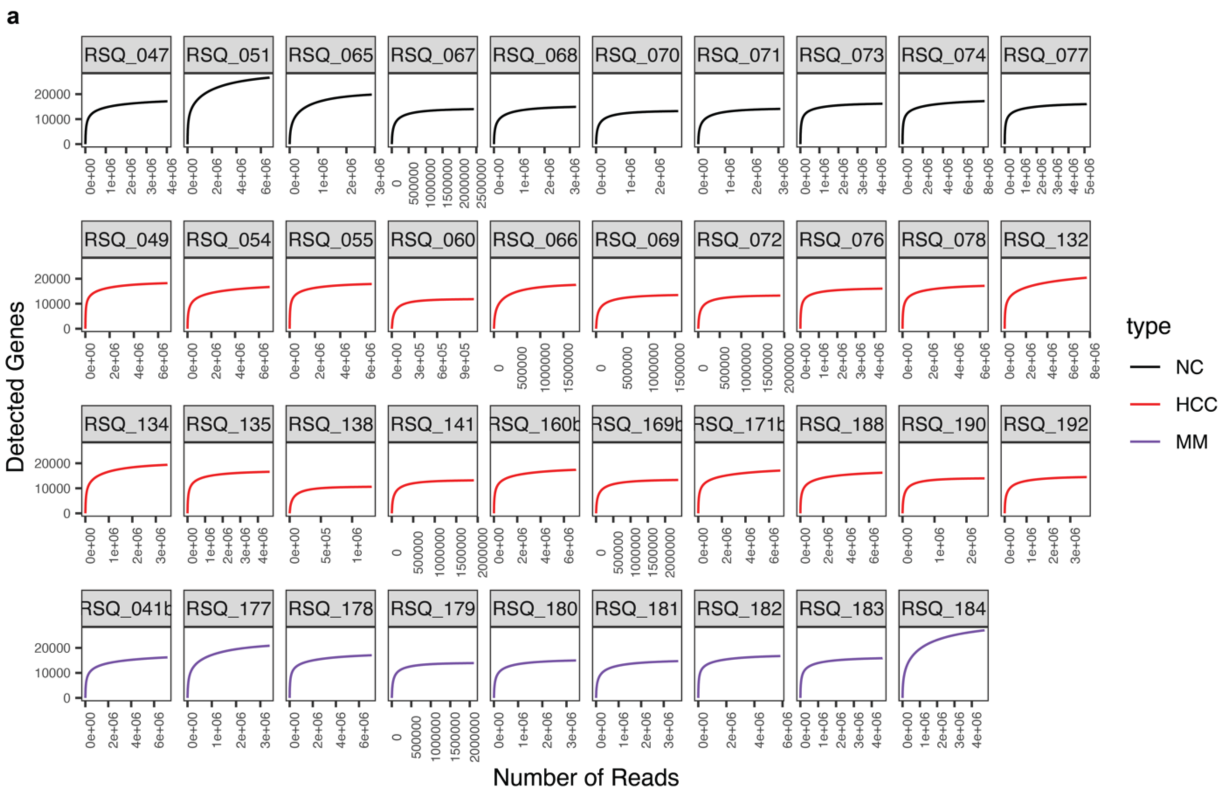

Supplementary Figure 6 | Sequencing saturation analysis of all 39 samples in the independent validation cohort. Total reads for each sample were subsampled (x-axis) and the number of genes detected at that read depth is reported (y-axis).

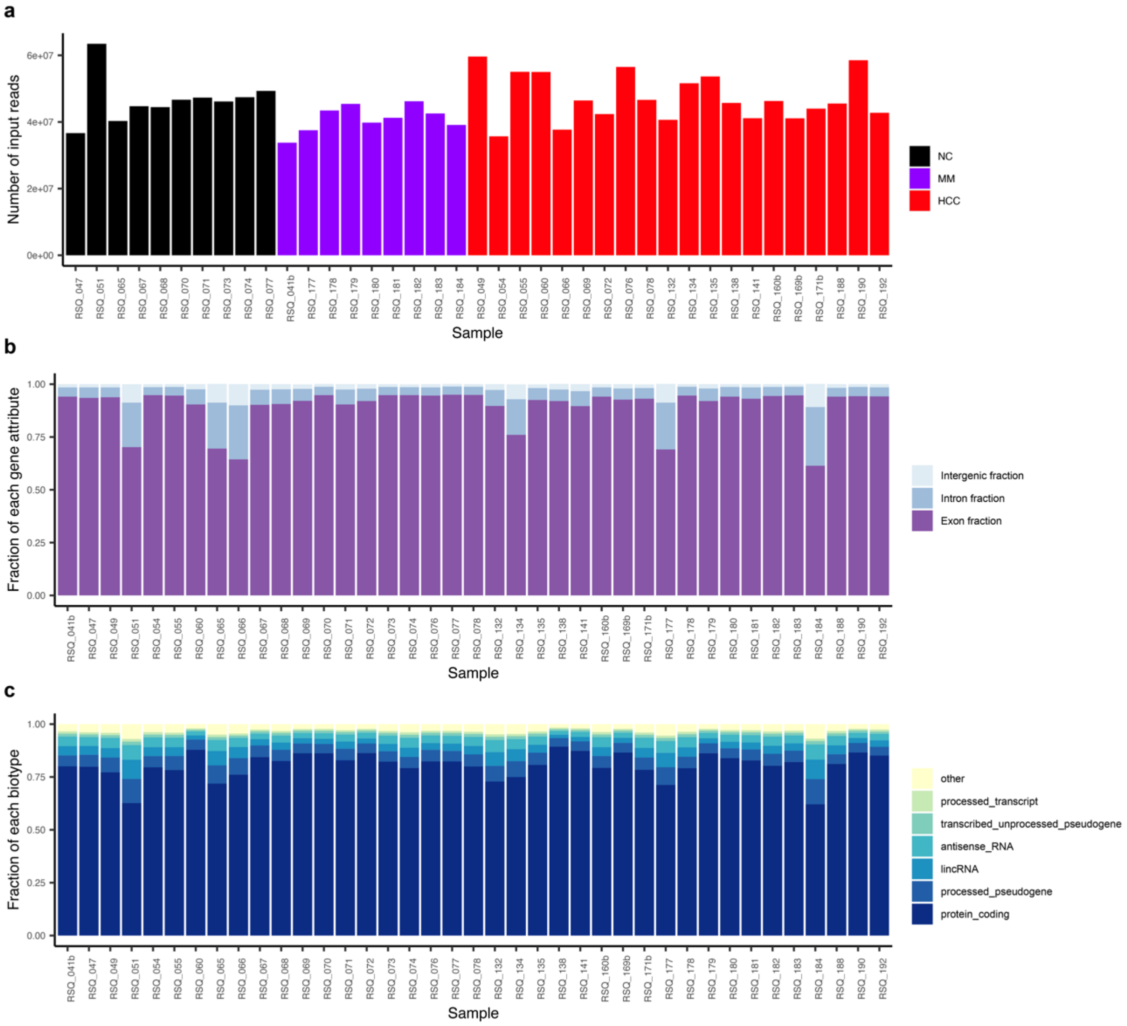

Supplementary Figure 7 | Distribution of (**a**) sequencing reads, (**b**) exon/intron and intergenic fractions, and (**c**) coverage of the transcriptome across all 39 samples in the independent validation cohort.

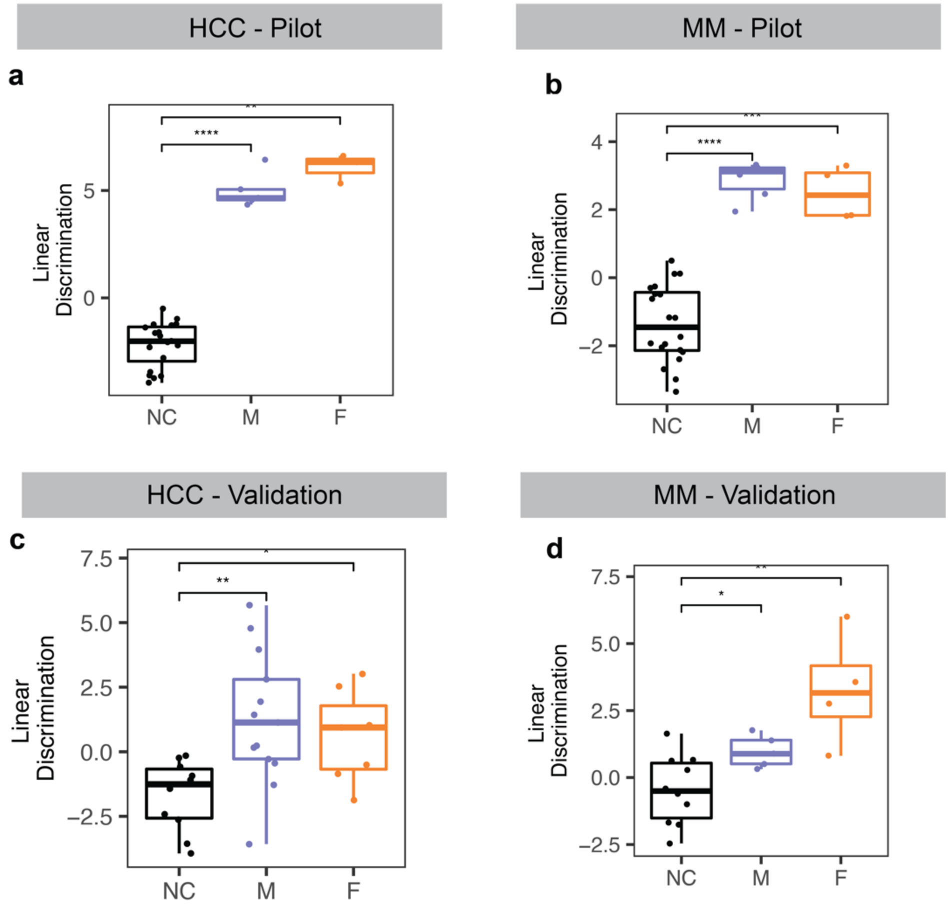

Supplementary Figure 8 | Linear Discriminant Analysis (LDA) across males and females in HCC and MM in our pilot cohort (**a, b**). Using the models trained on our pilot cohort, we also tested discrimination by LDA in our external validation set for both HCC and MM across males and females (**c, d**). P-values for each pair are calculated by the Wilcoxon rank sum test. Centre-line indicates the median value across all patients in that group, and the hinges represent the lower (Q1) and upper (Q3) quartile, with whiskers extending to the minimum and maximum of the resulting distribution.

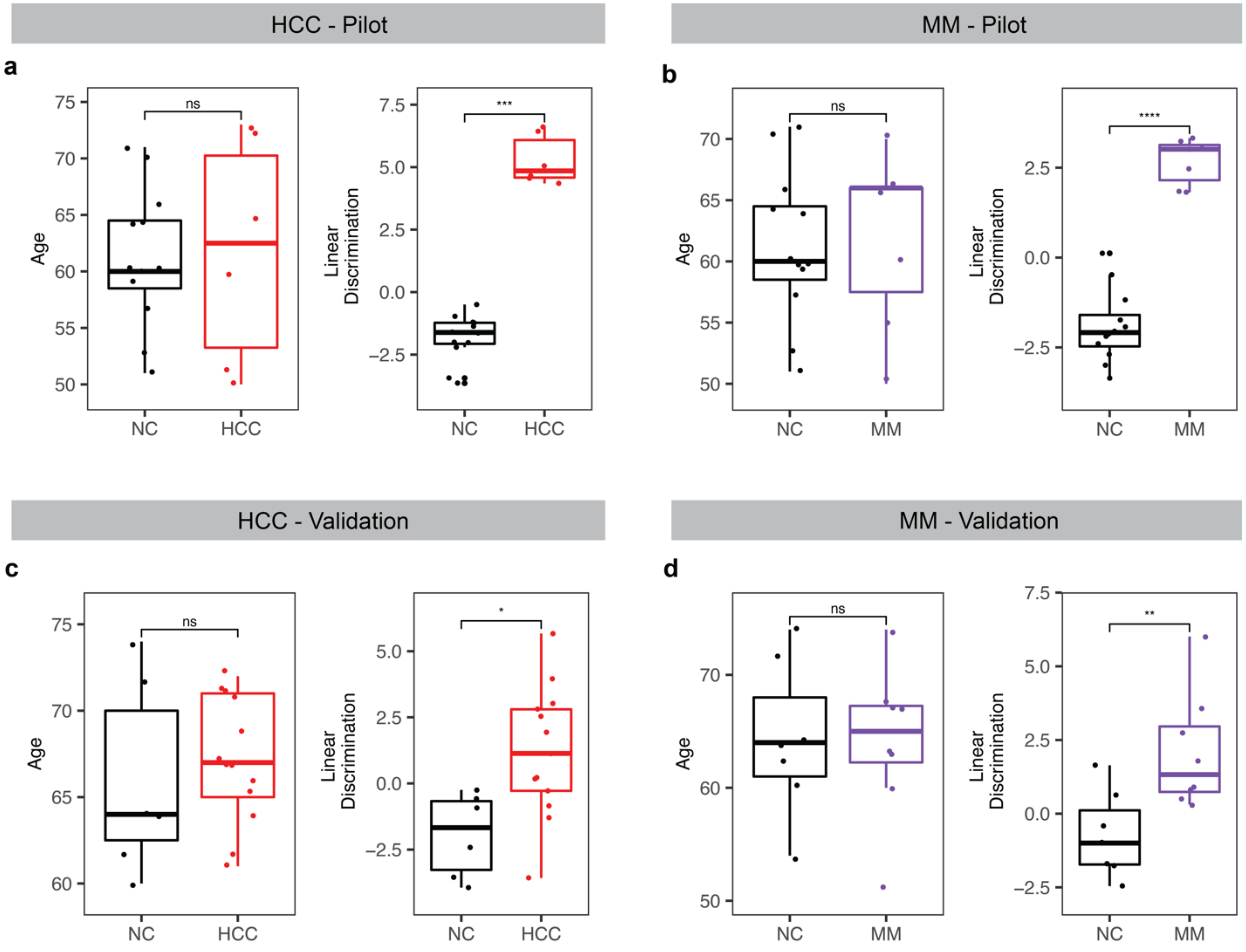

Supplementary Figure 9 | Linear Discriminant for the subset of the samples in which cases and controls had comparable median age and standard deviation. In this reduced sample set, we show that when a difference in age between cases and controls is not significant, discrimination by Linear Discriminant Analysis (LDA) is still significant. This was done with the models trained on our pilot cohort, and tested for each pairwise comparison of NC – HCC (**a**), NC – MM (**b**) in our pilot cohort, and our validation cohort (**c,d**). P-values for each pair are calculated by the Wilcoxon rank sum test. Centre-line indicates the median value across all patients in that group, and the hinges represent the lower (Q1) and upper (Q3) quartile, with whiskers extending to the minimum and maximum of the resulting distribution.

Supplementary Tables

Supplementary Table 1 | Clinical Information for the pilot sample set and validation sample set

Set	SeqID	Age	Sex	Group	Stage	Etiology	Race
Pilot	PP027	73	M	Liver Cancer	C	na	WHITE
Pilot	PP099	72	M	Liver Cancer	B	Hemochromatosis, jaundice, Gilberts disease	WHITE
Pilot	PP048	60	F	Liver Cancer	D	NASH	WHITE
Pilot	PP026	65	M	Liver Cancer	A	NASH	MORE THAN ONE RACE
Pilot	PP031	51	M	Liver Cancer	A	HCV+	WHITE
Pilot	PP109	75	F	Liver Cancer	C	NASH, Cirrhosis	WHITE
Pilot	PP050	82	F	Liver Cancer	A	NASH	WHITE
Pilot	PP111	50	M	Liver Cancer	A	NASH	WHITE
Pilot	PP078	59	F	Liver Cirrhosis	na	Cirrhosis secondary to sarcoidosis, HCV-/HBV-	WHITE
Pilot	PP061	50	F	Liver Cirrhosis	na	HCV+, Hepatitis	WHITE
Pilot	PP035	48	F	Liver Cirrhosis	na	Hepatitis	WHITE
Pilot	PP075	33	M	Liver Cirrhosis	na	Budd Chiari	WHITE
Pilot	PP073	66	M	Multiple Myeloma	I	na	WHITE
Pilot	PP010	66	M	Multiple Myeloma	II	na	WHITE
Pilot	PP008	70	F	Multiple Myeloma	II	na	WHITE
Pilot	PP047	31	M	Multiple Myeloma	II	na	WHITE
Pilot	PP015	66	F	Multiple Myeloma	III	na	WHITE
Pilot	PP074	35	F	Multiple Myeloma	II	na	WHITE
Pilot	PP098	55	M	Multiple Myeloma	II	na	WHITE
Pilot	PP005	60	M	Multiple Myeloma	II	na	WHITE
Pilot	PP007	50	F	Multiple Myeloma	I	na	WHITE
Pilot	PP049	73	M	Multiple Myeloma	III	na	WHITE
Pilot	PP097	64	F	MGUS	na	na	WHITE
Pilot	PP018	73	M	MGUS	na	na	WHITE
Pilot	PP060	68	M	MGUS	na	na	WHITE
Pilot	PP036	57	M	MGUS	na	na	WHITE
Pilot	PP114	70	F	MGUS	na	na	WHITE
Pilot	PP103	56	F	MGUS	na	na	WHITE
Pilot	PP058	67	M	MGUS	na	na	WHITE
Pilot	PP017	64	M	MGUS	na	na	WHITE
Pilot	PP102	47	M	Non-Cancer	na	na	WHITE
Pilot	PP055	37	F	Non-Cancer	na	na	WHITE
Pilot	PP019	59	F	Non-Cancer	na	na	WHITE
Pilot	PP032	57	M	Non-Cancer	na	na	WHITE
Pilot	PP062	71	F	Non-Cancer	na	na	WHITE
Pilot	PP011	60	F	Non-Cancer	na	na	WHITE
Pilot	PP041	64	F	Non-Cancer	na	na	WHITE
Pilot	PP004	46	F	Non-Cancer	na	na	WHITE
Pilot	PP003	42	M	Non-Cancer	na	na	ASIAN
Pilot	PP105	43	F	Non-Cancer	na	na	WHITE
Pilot	PP012	30	M	Non-Cancer	na	na	WHITE
Pilot	PP046	53	F	Non-Cancer	na	na	WHITE
Pilot	PP087	41	F	Non-Cancer	na	na	WHITE
Pilot	PP016	66	F	Non-Cancer	na	na	WHITE
Pilot	PP029	51	M	Non-Cancer	na	na	ASIAN
Pilot	PP043	42	M	Non-Cancer	na	na	WHITE
Pilot	PP020	60	F	Non-Cancer	na	na	WHITE
Pilot	PP002	64	F	Non-Cancer	na	na	WHITE
Pilot	PP116	60	M	Non-Cancer	na	na	WHITE
Pilot	PP091	70	M	Non-Cancer	na	na	WHITE

Validation	RSQ_072	76	F	Liver Cancer	A	HCV successfully treated	WHITE
Validation	RSQ_066	72	F	Liver Cancer	B	HCV +	WHITE
Validation	RSQ_054	81	F	Liver Cancer	B	HCV+	WHITE
Validation	RSQ_060	69	M	Liver Cancer	A	HCV+	WHITE
Validation	RSQ_076	61	M	Liver Cancer	B	HCV+	WHITE
Validation	RSQ_078	67	M	Liver Cancer	A	HCV+	WHITE
Validation	RSQ_069	62	M	Liver Cancer	B	HCV+	WHITE
Validation	RSQ_049	67	M	Liver Cancer	A	HCV+	WHITE
Validation	RSQ_055	64	F	Liver Cancer	B	Cirrhosis (HCV-, HBV-)	WHITE
Validation	RSQ_134	65	F	Liver Cancer	A	HCV+	WHITE
Validation	RSQ_160b	71	M	Liver Cancer	B	HCV+	WHITE
Validation	RSQ_135	47	M	Liver Cancer	B	HCV+	WHITE
Validation	RSQ_171b	71	M	Liver Cancer	A	HCV+	WHITE
Validation	RSQ_169b	66	M	Liver Cancer	A	HCV+	WHITE
Validation	RSQ_138	78	F	Liver Cancer	B	not documented in EMR	WHITE
Validation	RSQ_141	71	M	Liver Cancer	B	HCV+	WHITE
Validation	RSQ_132	76	M	Liver Cancer	A	Hemochromatosis, jaundice, Gilbert disease	WHITE
Validation	RSQ_188	82	M	Liver Cancer	C	Fatty Liver	WHITE
Validation	RSQ_190	67	M	Liver Cancer	A	HCV+	WHITE
Validation	RSQ_192	78	F	Liver Cancer	B	Alcoholic cirrhosis and steatoheaptitis	WHITE
Validation	RSQ_177	68	M	Multiple Myeloma	I	na	WHITE
Validation	RSQ_178	67	M	Multiple Myeloma	II	na	WHITE
Validation	RSQ_179	63	F	Multiple Myeloma	II	na	WHITE
Validation	RSQ_180	74	F	Multiple Myeloma	III	na	WHITE
Validation	RSQ_181	63	M	Multiple Myeloma	I	na	WHITE
Validation	RSQ_182	28	M	Multiple Myeloma	I	na	WHITE
Validation	RSQ_183	60	F	Multiple Myeloma	III	na	WHITE
Validation	RSQ_184	67	F	Multiple Myeloma	I	na	WHITE
Validation	RSQ_041b	51	M	Multiple Myeloma	I	na	WHITE
Validation	RSQ_067	72	M	Non-Cancer	na	na	WHITE
Validation	RSQ_070	62	M	Non-Cancer	na	na	WHITE
Validation	RSQ_077	64	F	Non-Cancer	na	na	WHITE
Validation	RSQ_065	74	F	Non-Cancer	na	na	WHITE
Validation	RSQ_073	25	F	Non-Cancer	na	na	WHITE
Validation	RSQ_051	60	F	Non-Cancer	na	na	WHITE
Validation	RSQ_074	54	F	Non-Cancer	na	na	WHITE
Validation	RSQ_071	21	M	Non-Cancer	na	na	WHITE
Validation	RSQ_068	64	F	Non-Cancer	na	na	WHITE
Validation	RSQ_047	27	F	Non-Cancer	na	na	ASIAN

*Abbreviation

NASH : Non-alcoholic steatohepatitis

HCV : Hepatitis C virus

HBV : Hepatitis B virus

EMR: Electronic medical record

Supplementary Table 2 | Table of sequencing reads, unique reads, exon/intron/intergenic fractions, and fraction of protein coding across all 89 samples from the pilot sample set and independent validation set prepared in different library batches

Set	SeqID	RNA Extraction	Library Preparation	Total Number of Reads	Number of Unique Reads	Exon Fraction	Intron Fraction	Intergenic Fraction	Fraction Protein Coding
Pilot	PP002	batch 1	batch 1	33192487	9477957	0.93	0.05	0.02	0.81
Pilot	PP003	batch 1	batch 1	29467052	4214981	0.86	0.11	0.03	0.82
Pilot	PP004	batch 1	batch 1	29574675	3825380	0.82	0.13	0.04	0.85
Pilot	PP005	batch 1	batch 1	33768635	4088109	0.86	0.1	0.03	0.83
Pilot	PP007	batch 1	batch 1	31698177	1171491	0.84	0.13	0.03	0.89
Pilot	PP008	batch 1	batch 1	30079806	10677465	0.74	0.19	0.07	0.74
Pilot	PP010	batch 2	batch 1	38503355	3863232	0.76	0.18	0.06	0.81
Pilot	PP011	batch 2	batch 1	31034252	4395327	0.86	0.11	0.04	0.84
Pilot	PP012	batch 2	batch 1	35799309	4766786	0.92	0.07	0.02	0.83
Pilot	PP015	batch 2	batch 1	30093097	6852599	0.84	0.12	0.04	0.87
Pilot	PP016	batch 2	batch 1	29890017	6853766	0.86	0.1	0.04	0.81
Pilot	PP017	batch 3	batch 1	34646834	6092611	0.69	0.23	0.08	0.77
Pilot	PP018	batch 3	batch 1	35833774	3469637	0.59	0.3	0.1	0.81
Pilot	PP019	batch 3	batch 1	36506970	4711445	0.72	0.21	0.07	0.76
Pilot	PP020	batch 3	batch 1	34201040	5321020	0.8	0.15	0.05	0.76
Pilot	PP026	batch 4	batch 1	40104631	3473624	0.83	0.13	0.05	0.84
Pilot	PP027	batch 4	batch 1	34476121	17271358	0.85	0.11	0.04	0.74
Pilot	PP029	batch 4	batch 1	30273014	3882069	0.9	0.08	0.02	0.84
Pilot	PP031	batch 4	batch 1	35139077	9791729	0.7	0.25	0.06	0.76
Pilot	PP032	batch 4	batch 1	31904022	4016176	0.87	0.1	0.04	0.83
Pilot	PP035	batch 4	batch 2	30996985	13700958	0.86	0.11	0.04	0.78
Pilot	PP036	batch 4	batch 2	36919622	7006298	0.71	0.22	0.07	0.8
Pilot	PP041	batch 5	batch 2	35773928	6240377	0.83	0.13	0.04	0.82
Pilot	PP043	batch 5	batch 2	31753026	7231832	0.71	0.22	0.07	0.81
Pilot	PP046	batch 5	batch 2	30298636	4380430	0.84	0.13	0.04	0.84
Pilot	PP047	batch 5	batch 2	31468616	13876029	0.93	0.05	0.02	0.81
Pilot	PP048	batch 5	batch 2	32642636	8534075	0.82	0.14	0.05	0.81
Pilot	PP049	batch 6	batch 2	37084139	9845748	0.88	0.08	0.03	0.89
Pilot	PP050	batch 6	batch 2	40532989	5076857	0.87	0.1	0.03	0.87
Pilot	PP055	batch 6	batch 2	34851176	15323436	0.91	0.07	0.02	0.83
Pilot	PP058	batch 7	batch 2	36866771	5758608	0.9	0.08	0.02	0.85
Pilot	PP060	batch 7	batch 2	37126236	4991733	0.75	0.19	0.06	0.82
Pilot	PP061	batch 7	batch 2	37509562	13009859	0.78	0.18	0.03	0.78
Pilot	PP062	batch 7	batch 2	36883791	21874141	0.9	0.08	0.03	0.83
Pilot	PP073	batch 8	batch 3	43566479	2482378	0.8	0.16	0.04	0.87
Pilot	PP074	batch 8	batch 3	34208260	5631056	0.92	0.06	0.02	0.85
Pilot	PP075	batch 8	batch 3	31957614	5211761	0.84	0.12	0.04	0.84
Pilot	PP078	batch 8	batch 3	36596920	14457187	0.89	0.08	0.03	0.78
Pilot	PP087	batch 9	batch 3	34570617	2699261	0.8	0.15	0.05	0.84
Pilot	PP091	batch 10	batch 3	38121200	3181309	0.82	0.14	0.04	0.83
Pilot	PP097	batch 11	batch 4	27998299	3867357	0.81	0.15	0.05	0.84
Pilot	PP098	batch 11	batch 4	27748334	14243418	0.68	0.24	0.08	0.75
Pilot	PP099	batch 11	batch 4	28514250	6664874	0.77	0.18	0.06	0.8
Pilot	PP102	batch 11	batch 4	28850027	7551982	0.89	0.09	0.03	0.83
Pilot	PP103	batch 11	batch 4	27808286	14620805	0.45	0.4	0.15	0.65
Pilot	PP105	batch 11	batch 4	30350083	8031052	0.83	0.13	0.04	0.82
Pilot	PP109	batch 12	batch 4	28245277	14323721	0.71	0.21	0.08	0.74
Pilot	PP111	batch 12	batch 4	33349408	7413209	0.77	0.17	0.06	0.83
Pilot	PP114	batch 12	batch 4	33053778	2478571	0.73	0.2	0.06	0.86
Pilot	PP116	batch 12	batch 4	31973526	4755722	0.87	0.11	0.03	0.85
Validation	RSQ_041b	batch 4	batch 3	33772810	29487019	0.94	0.04	0.02	0.80
Validation	RSQ_047	batch 1	batch 1	36641798	19005596	0.94	0.05	0.02	0.80
Validation	RSQ_049	batch 1	batch 2	59627326	40642737	0.94	0.05	0.02	0.77
Validation	RSQ_051	batch 1	batch 2	63448881	44393036	0.70	0.21	0.09	0.63
Validation	RSQ_054	batch 1	batch 2	35688068	29607149	0.95	0.04	0.01	0.80
Validation	RSQ_055	batch 1	batch 2	55042717	38897839	0.95	0.04	0.01	0.78
Validation	RSQ_060	batch 5	batch 2	54988336	24189119	0.90	0.07	0.02	0.88
Validation	RSQ_065	batch 2	batch 2	40294719	31024201	0.70	0.22	0.09	0.72
Validation	RSQ_066	batch 2	batch 2	37682907	24430096	0.64	0.26	0.10	0.76
Validation	RSQ_067	batch 2	batch 2	44706577	33873748	0.90	0.07	0.03	0.84
Validation	RSQ_068	batch 2	batch 2	44448294	35928995	0.91	0.07	0.02	0.83
Validation	RSQ_069	batch 2	batch 2	46440958	11272976	0.92	0.06	0.02	0.86
Validation	RSQ_070	batch 2	batch 2	46662511	37811541	0.95	0.04	0.01	0.86
Validation	RSQ_071	batch 2	batch 2	47314236	39458883	0.90	0.07	0.03	0.83
Validation	RSQ_072	batch 2	batch 2	42356958	31455839	0.92	0.06	0.02	0.86
Validation	RSQ_073	batch 3	batch 2	46146349	37190739	0.95	0.04	0.01	0.82
Validation	RSQ_074	batch 3	batch 2	47427477	41824062	0.95	0.04	0.01	0.79
Validation	RSQ_076	batch 3	batch 2	56514756	45112223	0.95	0.04	0.02	0.82
Validation	RSQ_077	batch 3	batch 2	49317771	41506358	0.95	0.04	0.01	0.82
Validation	RSQ_078	batch 3	batch 2	46629655	36123700	0.95	0.04	0.01	0.80
Validation	RSQ_132	batch 6	batch 5	40631324	33192598	0.90	0.08	0.03	0.73
Validation	RSQ_134	batch 6	batch 5	51608369	31562510	0.76	0.17	0.07	0.75
Validation	RSQ_135	batch 6	batch 5	53647921	38218341	0.93	0.06	0.02	0.81

Validation	RSQ_138	batch 7	batch 5	45699045	40021174	0.92	0.06	0.03	0.89
Validation	RSQ_141	batch 7	batch 5	41127111	34182473	0.90	0.07	0.03	0.87
Validation	RSQ_160b	batch 8	batch 6	46286176	37326907	0.94	0.04	0.02	0.79
Validation	RSQ_169b	batch 7	batch 3	41091726	34452215	0.93	0.05	0.02	0.87
Validation	RSQ_171b	batch 6	batch 3	43997417	35073332	0.93	0.05	0.02	0.78
Validation	RSQ_177	batch 4	batch 4	37506679	30534084	0.69	0.22	0.09	0.71
Validation	RSQ_178	batch 4	batch 4	43446571	36566835	0.95	0.04	0.01	0.79
Validation	RSQ_179	batch 4	batch 4	45419463	35887750	0.92	0.06	0.02	0.86
Validation	RSQ_180	batch 4	batch 4	39794385	31445458	0.94	0.05	0.01	0.84
Validation	RSQ_181	batch 4	batch 4	41258803	34374819	0.93	0.05	0.02	0.83
Validation	RSQ_182	batch 4	batch 4	46219892	39711701	0.94	0.04	0.01	0.80
Validation	RSQ_183	batch 4	batch 4	42546602	34105678	0.95	0.04	0.01	0.82
Validation	RSQ_184	batch 4	batch 4	39110078	33476255	0.61	0.28	0.11	0.62
Validation	RSQ_188	batch 9	batch 4	45529234	40591516	0.94	0.04	0.02	0.81
Validation	RSQ_190	batch 9	batch 4	58526837	47914592	0.94	0.04	0.01	0.87
Validation	RSQ_192	batch 9	batch 4	42744939	37325748	0.94	0.04	0.02	0.85

Supplementary Table 3 | Differential expression analysis of pairwise comparison between individual cancer or precancerous condition with respect to non-cancer using DESeq2 (adjusted p-value < 0.01)

Pair	gene	baseMean	log2FoldChange	lfcSE	stat	pvalue	padj
HCC vs NC	FGB	9.54043945	2.383146669	0.304651	7.48821514	6.98E-14	2.09E-09
HCC vs NC	HRG	18.79151349	2.317610669	0.307693	7.196784468	6.16E-13	9.22E-09
HCC vs NC	FGA	10.83667317	2.248298714	0.304322	7.114194336	1.13E-12	1.12E-08
HCC vs NC	C3	18.08752279	1.693868459	0.287793	5.775592922	7.67E-09	5.73E-05
HCC vs NC	FGG	6.074544421	1.831111187	0.311824	5.670245138	1.43E-08	8.53E-05
HCC vs NC	CP	3.568402572	1.78771012	0.309279	5.570509436	2.54E-08	9.49E-05
HCC vs NC	APOH	13.60227841	1.675855895	0.294434	5.5726422	2.51E-08	9.49E-05
HCC vs NC	HULC	2.162614188	1.845224079	0.314827	5.589816874	2.27E-08	9.49E-05
HCC vs NC	IFITM3	177.2018786	1.408295495	0.2583	5.396814888	6.78E-08	0.000225
HCC vs NC	UGT2B15	2.739636172	1.546025091	0.298857	5.019417165	5.18E-07	0.001549
HCC vs NC	VTN	4.420716817	1.558599628	0.314984	4.742574033	2.11E-06	0.005735
HCC vs NC	CAPRIN2	19.7058533	0.853553652	0.182862	4.671994317	2.98E-06	0.007431
MM vs NC	CENPE	48.17985185	1.144883511	0.152972	7.486721412	7.06E-14	2.06E-09
MM vs NC	CPOX	25.93497038	1.395128166	0.200926	6.944051494	3.81E-12	3.70E-08
MM vs NC	NUSAP1	33.50897048	1.234770336	0.177095	6.979509341	2.96E-12	3.70E-08
MM vs NC	CA1	397.5266752	1.722231932	0.25239	6.800343407	1.04E-11	7.60E-08
MM vs NC	NEK2	4.616667065	2.012818527	0.303188	6.534030498	6.40E-11	3.73E-07
MM vs NC	HBD	109.7044137	1.606600785	0.248832	6.439310252	1.20E-10	5.82E-07
MM vs NC	HBG2	312.1812688	2.348168416	0.359616	6.330390807	2.45E-10	1.02E-06
MM vs NC	AHSP	46.95512597	1.528009653	0.248133	6.148223471	7.84E-10	2.85E-06
MM vs NC	ELL2	21.91988323	1.344413464	0.220952	6.084177716	1.17E-09	2.92E-06
MM vs NC	MKI67	83.76610812	1.265283784	0.207217	6.102320006	1.05E-09	2.92E-06
MM vs NC	AGL	30.73272804	1.505269004	0.247266	6.074314218	1.25E-09	2.92E-06
MM vs NC	EPB42	57.47809392	1.551699089	0.253732	6.100135774	1.06E-09	2.92E-06
MM vs NC	HBG1	112.8246854	2.300701344	0.366232	6.067148729	1.30E-09	2.92E-06
MM vs NC	SMC4	116.8891212	0.738932852	0.122814	6.017945964	1.77E-09	3.67E-06
MM vs NC	ASPM	25.99235817	1.343802169	0.224609	5.983071971	2.19E-09	4.25E-06
MM vs NC	NUDT4	112.9786718	1.180281587	0.206412	5.714481087	1.10E-08	2.00E-05
MM vs NC	PSME4	129.6888427	1.160360801	0.208525	5.560476508	2.69E-08	4.61E-05
MM vs NC	GYPA	69.35867145	1.445922865	0.25991	5.548700732	2.88E-08	4.65E-05
MM vs NC	KAT2B	262.1259581	1.376488595	0.249432	5.504032712	3.71E-08	5.39E-05
MM vs NC	ABCB10	41.31134237	1.396088965	0.252814	5.512090295	3.55E-08	5.39E-05
MM vs NC	ENTPD5	26.59105754	1.717036186	0.309655	5.495693776	3.89E-08	5.39E-05
MM vs NC	CD96	33.6920111	1.740101597	0.3158	5.448193767	5.09E-08	6.73E-05
MM vs NC	SPTA1	95.98507999	1.277217476	0.236548	5.391625625	6.98E-08	8.84E-05
MM vs NC	TFRC	99.8315754	1.156604055	0.216347	5.343871712	9.10E-08	0.00011
MM vs NC	BBOF1	102.2996997	1.323527358	0.2483	5.319371668	1.04E-07	0.000121
MM vs NC	AIDA	93.76039802	0.700482445	0.132373	5.292685612	1.21E-07	0.000135
MM vs NC	IFIT1B	62.93158945	1.462521801	0.276723	5.263410008	1.41E-07	0.000152
MM vs NC	CCNA2	8.926517153	1.471093497	0.285828	5.148513154	2.63E-07	0.000273
MM vs NC	TFDP1	192.2094247	1.416020274	0.276331	5.101798841	3.36E-07	0.000326
MM vs NC	HBM	36.38217871	1.31495253	0.257367	5.102997052	3.34E-07	0.000326
MM vs NC	USP7	155.0058717	0.653885398	0.128764	5.079074351	3.79E-07	0.000356
MM vs NC	SLC6A8	29.56748222	1.612394482	0.315417	5.070486199	3.97E-07	0.000359
MM vs NC	ALAS2	406.6065512	1.600394651	0.313128	5.059922663	4.19E-07	0.000359
MM vs NC	OSBP2	212.0418408	1.498884226	0.294226	5.060561911	4.18E-07	0.000359
MM vs NC	UBB	920.1466897	0.744822366	0.147424	5.051848057	4.38E-07	0.000364
MM vs NC	RAP1GAP	7.154304682	1.948817497	0.372504	5.040172452	4.65E-07	0.000376
MM vs NC	GAB1	46.98928125	0.946297508	0.187896	5.034762379	4.78E-07	0.000376

MM vs NC	ZNF385D	56.25431831	-1.649886457	0.334212	-5.021590136	5.12E-07	0.000393
MM vs NC	MYBL2	32.66205417	1.331210402	0.264978	5.005465358	5.57E-07	0.000406
MM vs NC	HBE1	9.084378536	1.869498382	0.364036	5.009707027	5.45E-07	0.000406
MM vs NC	TMEM14B	26.3168985	1.004927996	0.202273	4.969204011	6.72E-07	0.000477
MM vs NC	TMEM56	33.16949522	1.310236151	0.2642	4.953422664	7.29E-07	0.000505
MM vs NC	HIST1H1B	68.67388896	1.091995954	0.220934	4.939332953	7.84E-07	0.000531
MM vs NC	GYPC	201.1491307	1.077350509	0.223248	4.821367024	1.43E-06	0.000943
MM vs NC	IFI27	41.9367921	1.530523428	0.315312	4.809833729	1.51E-06	0.000977
MM vs NC	SOX6	46.50591066	1.207213296	0.251325	4.798381997	1.60E-06	0.000991
MM vs NC	FCGR2A	85.78951546	1.485128883	0.306855	4.799032138	1.59E-06	0.000991
MM vs NC	TMOD1	59.93886672	1.019369045	0.213882	4.765453096	1.88E-06	0.001143
MM vs NC	CREB3L1	14.35800377	1.594848362	0.347123	4.730732815	2.24E-06	0.001329
MM vs NC	PRDX2	33.03810926	1.160061025	0.246038	4.709081177	2.49E-06	0.001449
MM vs NC	RSAD2	39.74329843	1.12960219	0.241446	4.677795107	2.90E-06	0.001655
MM vs NC	CENPF	260.3829103	1.207317796	0.258951	4.649348614	3.33E-06	0.001864
MM vs NC	HMGB2	285.4058456	0.55205218	0.119428	4.622764274	3.79E-06	0.00208
MM vs NC	OAT	27.97001255	0.925873663	0.201972	4.584834298	4.54E-06	0.002449
MM vs NC	SDC4	28.44871687	-1.540713291	0.344329	-4.572486359	4.82E-06	0.002455
MM vs NC	ST6GALNAC4	13.96679824	1.178040605	0.257751	4.572888545	4.81E-06	0.002455
MM vs NC	ITGB3BP	76.46566185	-1.153878384	0.252465	-4.568252228	4.92E-06	0.002455
MM vs NC	PHOSPHO1	38.80438161	1.219476219	0.266689	4.565757106	4.98E-06	0.002455
MM vs NC	PRC1	17.12829389	1.022540788	0.223562	4.576389813	4.73E-06	0.002455
MM vs NC	HSD17B4	67.99839238	0.515489438	0.113759	4.532992822	5.82E-06	0.002821
MM vs NC	CDH1	5.633976149	1.493656168	0.326341	4.522846714	6.10E-06	0.002911
MM vs NC	PTK7	11.21095149	-1.567281921	0.369238	-4.503992264	6.67E-06	0.003131
MM vs NC	TPX2	19.51869751	1.023492939	0.227436	4.499137419	6.82E-06	0.003152
MM vs NC	CDK1	10.25220254	1.139522542	0.255286	4.474764811	7.65E-06	0.003479
MM vs NC	SLC25A39	154.5738156	1.154106148	0.257935	4.464307809	8.03E-06	0.003543
MM vs NC	TOP2A	36.19644848	0.897291219	0.201017	4.466482024	7.95E-06	0.003543
MM vs NC	TSPYL2	45.71344261	1.093161035	0.245057	4.455018652	8.39E-06	0.003644
MM vs NC	PPP1CB	274.1173484	0.680601249	0.153927	4.421234884	9.81E-06	0.004201
MM vs NC	ARG1	21.02100374	1.25925184	0.285379	4.402402499	1.07E-05	0.004516
MM vs NC	MITD1	137.2569773	-0.801823857	0.18248	-4.393960127	1.11E-05	0.004538
MM vs NC	ELOVL6	5.638687321	1.235211734	0.280975	4.392739902	1.12E-05	0.004538
MM vs NC	KEL	6.82920782	1.312061972	0.297877	4.392145889	1.12E-05	0.004538
MM vs NC	SLC38A5	14.71133594	1.231965985	0.28052	4.385157009	1.16E-05	0.004559
MM vs NC	SOCS2	21.85434465	1.128616205	0.256735	4.386448461	1.15E-05	0.004559
MM vs NC	LGALS3	85.63099415	0.773452672	0.177256	4.366099205	1.26E-05	0.004844
MM vs NC	LINC01781	4.065055803	-1.618823759	0.370861	-4.367305996	1.26E-05	0.004844
MM vs NC	CISD2	64.32206286	1.140611121	0.261771	4.34807704	1.37E-05	0.005192
MM vs NC	HPS1	65.19810328	0.785210199	0.180813	4.343062644	1.41E-05	0.005244
MM vs NC	C9orf40	18.50369144	1.401356403	0.320284	4.338460561	1.43E-05	0.005287
MM vs NC	ZNF542P	128.7646165	-1.418097998	0.333639	-4.328711363	1.50E-05	0.005457
MM vs NC	WNK1	828.4506104	0.874942514	0.202978	4.307710665	1.65E-05	0.005657
MM vs NC	CRYM	8.319848528	1.667297124	0.371785	4.307381936	1.65E-05	0.005657
MM vs NC	HECTD2	14.3573525	-1.170675606	0.269229	-4.311272144	1.62E-05	0.005657
MM vs NC	FOXO4	74.92939478	0.820882448	0.190385	4.31327278	1.61E-05	0.005657
MM vs NC	ARHGAP11A	29.01664018	0.857005285	0.198808	4.31747447	1.58E-05	0.005657
MM vs NC	SAMD9	122.587491	0.821454042	0.190795	4.304650949	1.67E-05	0.005661
MM vs NC	WDR11-AS1	119.9053813	-1.376780688	0.325767	-4.286673436	1.81E-05	0.006068

MM vs NC	SKA1	2.46040502	1.631744419	0.369435	4.281870501	1.85E-05	0.00613
MM vs NC	YBX1	1213.47061	0.462254723	0.108266	4.269644919	1.96E-05	0.006263
MM vs NC	CTSB	134.6444462	0.974264083	0.227936	4.272190206	1.94E-05	0.006263
MM vs NC	ARMC3	25.47628363	-1.400115583	0.327944	-4.271500174	1.94E-05	0.006263
MM vs NC	RRM2	10.68177932	1.195474677	0.280142	4.263385109	2.01E-05	0.006302
MM vs NC	HBB	7282.040547	1.110560161	0.259698	4.26464549	2.00E-05	0.006302
MM vs NC	YBX3	556.1581858	0.894395866	0.209786	4.260368036	2.04E-05	0.00632
MM vs NC	CREG1	133.8111897	0.906388177	0.212979	4.254127913	2.10E-05	0.00643
MM vs NC	RHAG	15.65921616	1.282224529	0.300428	4.248028996	2.16E-05	0.006539
MM vs NC	MMP8	13.99073601	1.561964406	0.360793	4.233369769	2.30E-05	0.006908
MM vs NC	GRHL1	41.16232348	-1.448736175	0.35447	-4.225060058	2.39E-05	0.007095
MM vs NC	BLVRB	56.82702682	1.082675745	0.257618	4.198018279	2.69E-05	0.007838
MM vs NC	H3F3B	327.173554	-0.519226428	0.123673	-4.198191179	2.69E-05	0.007838
MM vs NC	HMMR	13.91332551	0.973499071	0.232528	4.193325949	2.75E-05	0.007845
MM vs NC	CMPK2	21.74447826	1.264338418	0.300044	4.195063494	2.73E-05	0.007845
MM vs NC	SLC4A1	550.611478	1.152463857	0.274393	4.184964937	2.85E-05	0.00806
MM vs NC	ALAD	43.97201475	0.754288665	0.180674	4.178216434	2.94E-05	0.008223
MM vs NC	LINC00989	368.9499221	-1.286938205	0.311552	-4.174752837	2.98E-05	0.00827
MM vs NC	KIF11	13.31781773	0.999792395	0.24017	4.165748658	3.10E-05	0.008522
MM vs NC	USP32	95.34787244	0.534980402	0.128713	4.157324881	3.22E-05	0.008759
MM vs NC	UROD	42.67164213	0.750573149	0.181193	4.145849797	3.39E-05	0.009125
MM vs NC	USP15	175.4236805	0.840247824	0.202991	4.137160504	3.52E-05	0.00939
MM vs NC	UBE2B	75.41679511	0.882883646	0.213737	4.129313001	3.64E-05	0.009628
Cirr vs NC	AGL	33.02779405	2.049587079	0.32065	6.327377054	2.49E-10	6.96E-06
Cirr vs NC	FCGR2A	86.81797865	2.05688026	0.327841	6.192870516	5.91E-10	8.24E-06
Cirr vs NC	RAP1GAP	8.560546486	2.833698078	0.438573	5.589457958	2.28E-08	0.000212
Cirr vs NC	OSBP2	233.2090641	2.082222621	0.367839	5.516218675	3.46E-08	0.000242
Cirr vs NC	GNS	22.17810447	1.505231445	0.284585	5.278076271	1.31E-07	0.000728
Cirr vs NC	LRP1	37.78641684	1.947514541	0.368454	5.165707804	2.40E-07	0.001114
Cirr vs NC	SCML1	5.436611454	2.094494958	0.396777	5.13415231	2.83E-07	0.00113
Cirr vs NC	IFITM3	145.5140589	1.507483216	0.310076	4.825563155	1.40E-06	0.004868
Cirr vs NC	TRAC	60.16050961	-1.509782361	0.316472	-4.775376961	1.79E-06	0.00556
Cirr vs NC	LEF1	53.65052484	-1.582519146	0.336445	-4.718630559	2.37E-06	0.006624
Cirr vs NC	CD3D	17.75869486	-1.776581903	0.373353	-4.680428666	2.86E-06	0.00726
Cirr vs NC	NSRP1	161.5807408	-0.708892776	0.151997	-4.66033095	3.16E-06	0.007326
Cirr vs NC	L3MBTL4	13.33743712	1.201778872	0.259447	4.644215836	3.41E-06	0.007326
Cirr vs NC	SYF2	231.3033469	-0.729634008	0.158187	-4.61048779	4.02E-06	0.007437
Cirr vs NC	PIK3R1	154.7487437	-0.840313031	0.183889	-4.565421758	4.98E-06	0.007437
Cirr vs NC	CD96	24.48352807	1.600009389	0.345904	4.571088362	4.85E-06	0.007437
Cirr vs NC	EIF1	1069.777812	1.134383481	0.248144	4.562082104	5.06E-06	0.007437
Cirr vs NC	METTL9	126.20809	0.934080489	0.202687	4.607296804	4.08E-06	0.007437
Cirr vs NC	TAPBP	43.35068267	0.980566406	0.214554	4.571537606	4.84E-06	0.007437
Cirr vs NC	RPL36A	198.4871465	-0.854627016	0.188111	-4.540818879	5.60E-06	0.007816
Cirr vs NC	DAP	477.4624866	1.656113341	0.36072	4.50385259	6.67E-06	0.008865
MGUS vs NC	TRADD	7.265471722	1.829025466	0.345767	5.289766229	1.22E-07	0.003718

Supplementary Table 4 | Results of permutation by random sample shuffling in each pair with 500 rounds between i) non-cancer and multiple myeloma and ii) non-cancer and hepatocellular carcinoma

Permutation	Number of genes with padj < 0.01
**Correct Assignment**	**110**
Permutation 1	0
Permutation 2	0
Permutation 3	0
Permutation 4	0
Permutation 5	0
Permutation 6	0
Permutation 7	0
Permutation 8	0
Permutation 9	0
Permutation 10	0
Permutation 11	0
Permutation 12	0
Permutation 13	0
Permutation 14	0
Permutation 15	0
Permutation 16	0
Permutation 17	0
Permutation 18	0
Permutation 19	0
Permutation 20	0
Permutation 21	0
Permutation 22	0
Permutation 23	0
Permutation 24	0
Permutation 25	0
Permutation 26	0
Permutation 27	0
Permutation 28	0
Permutation 29	0
Permutation 30	0
Permutation 31	0
Permutation 32	0
Permutation 33	0
Permutation 34	0
Permutation 35	0
Permutation 36	0
Permutation 37	0
Permutation 38	0
Permutation 39	0
Permutation 40	0
Permutation 41	0
Permutation 42	0
Permutation 43	0
Permutation 44	0
Permutation 45	0
Permutation 46	0
Permutation 47	0
Permutation 48	0
Permutation 49	0
Permutation 50	0
Permutation 51	0
Permutation 52	0
Permutation 53	0
Permutation 54	0
Permutation 55	0
Permutation 56	0
Permutation 57	0
Permutation 58	0
Permutation 59	0
Permutation 60	0
Permutation 61	0
Permutation 62	0
Permutation 63	0
Permutation 64	0
Permutation 65	0
Permutation 66	0
Permutation 67	0
Permutation 68	0
Permutation 69	0
Permutation 70	0
Permutation 71	0
Permutation 72	0
Permutation 73	0
Permutation 74	0
Permutation 75	0
Permutation 76	0
Permutation 77	0
Permutation 78	3
Permutation 79	0
Permutation 80	0
Permutation 81	0
Permutation 82	0
Permutation 83	0
Permutation 84	0
Permutation 85	0
Permutation 86	0
Permutation 87	0
Permutation 88	0
Permutation 89	0
Permutation 90	0
Permutation 91	0
Permutation 92	0
Permutation 93	0
Permutation 94	0
Permutation 95	0
Permutation 96	0
Permutation 97	0
Permutation 98	0
Permutation 99	0
Permutation 100	0

Permutation	Number of genes with padj < 0.01
Permutation 101	0
Permutation 102	0
Permutation 103	0
Permutation 104	0
Permutation 105	0
Permutation 106	0
Permutation 107	0
Permutation 108	0
Permutation 109	0
Permutation 110	0
Permutation 111	0
Permutation 112	0
Permutation 113	0
Permutation 114	3
Permutation 115	0
Permutation 116	0
Permutation 117	0
Permutation 118	0
Permutation 119	0
Permutation 120	0
Permutation 121	0
Permutation 122	0
Permutation 123	0
Permutation 124	0
Permutation 125	0
Permutation 126	0
Permutation 127	0
Permutation 128	0
Permutation 129	0
Permutation 130	0
Permutation 131	0
Permutation 132	0
Permutation 133	0
Permutation 134	2
Permutation 135	0
Permutation 136	0
Permutation 137	0
Permutation 138	0
Permutation 139	0
Permutation 140	0
Permutation 141	0
Permutation 142	0
Permutation 143	0
Permutation 144	0
Permutation 145	0
Permutation 146	0
Permutation 147	0
Permutation 148	1
Permutation 149	3
Permutation 150	0
Permutation 151	0
Permutation 152	0
Permutation 153	0
Permutation 154	0
Permutation 155	0
Permutation 156	0
Permutation 157	0
Permutation 158	0
Permutation 159	0
Permutation 160	0
Permutation 161	0
Permutation 162	0
Permutation 163	0
Permutation 164	0
Permutation 165	5
Permutation 166	0
Permutation 167	0
Permutation 168	0
Permutation 169	0
Permutation 170	0
Permutation 171	0
Permutation 172	0
Permutation 173	1
Permutation 174	0
Permutation 175	1
Permutation 176	0
Permutation 177	0
Permutation 178	0
Permutation 179	0
Permutation 180	0
Permutation 181	0
Permutation 182	0
Permutation 183	0
Permutation 184	0
Permutation 185	0
Permutation 186	0
Permutation 187	0
Permutation 188	0
Permutation 189	0
Permutation 190	0
Permutation 191	0
Permutation 192	0
Permutation 193	0
Permutation 194	0
Permutation 195	0
Permutation 196	0
Permutation 197	0
Permutation 198	0
Permutation 199	0
Permutation 200	0

Permutation	Number of genes with padj < 0.01
Permutation 201	0
Permutation 202	0
Permutation 203	0
Permutation 204	0
Permutation 205	0
Permutation 206	0
Permutation 207	0
Permutation 208	0
Permutation 209	0
Permutation 210	0
Permutation 211	0
Permutation 212	0
Permutation 213	0
Permutation 214	0
Permutation 215	0
Permutation 216	0
Permutation 217	0
Permutation 218	0
Permutation 219	0
Permutation 220	0
Permutation 221	1
Permutation 222	0
Permutation 223	0
Permutation 224	0
Permutation 225	0
Permutation 226	0
Permutation 227	0
Permutation 228	0
Permutation 229	0
Permutation 230	0
Permutation 231	0
Permutation 232	0
Permutation 233	1
Permutation 234	0
Permutation 235	0
Permutation 236	1
Permutation 237	0
Permutation 238	0
Permutation 239	0
Permutation 240	0
Permutation 241	0
Permutation 242	1
Permutation 243	0
Permutation 244	0
Permutation 245	0
Permutation 246	0
Permutation 247	0
Permutation 248	0
Permutation 249	0
Permutation 250	0
Permutation 251	0
Permutation 252	0
Permutation 253	0
Permutation 254	0
Permutation 255	0
Permutation 256	0
Permutation 257	0
Permutation 258	0
Permutation 259	0
Permutation 260	0
Permutation 261	0
Permutation 262	0
Permutation 263	0
Permutation 264	0
Permutation 265	0
Permutation 266	0
Permutation 267	0
Permutation 268	0
Permutation 269	0
Permutation 270	0
Permutation 271	0
Permutation 272	0
Permutation 273	0
Permutation 274	0
Permutation 275	0
Permutation 276	0
Permutation 277	0
Permutation 278	0
Permutation 279	0
Permutation 280	0
Permutation 281	0
Permutation 282	0
Permutation 283	0
Permutation 284	0
Permutation 285	0
Permutation 286	0
Permutation 287	0
Permutation 288	0
Permutation 289	0
Permutation 290	0
Permutation 291	0
Permutation 292	0
Permutation 293	0
Permutation 294	0
Permutation 295	0
Permutation 296	0
Permutation 297	0
Permutation 298	0
Permutation 299	0
Permutation 300	0

Permutation	Number of genes with padj < 0.01
Permutation 301	0
Permutation 302	0
Permutation 303	0
Permutation 304	0
Permutation 305	0
Permutation 306	0
Permutation 307	0
Permutation 308	0
Permutation 309	0
Permutation 310	0
Permutation 311	0
Permutation 312	0
Permutation 313	0
Permutation 314	1
Permutation 315	0
Permutation 316	0
Permutation 317	0
Permutation 318	0
Permutation 319	0
Permutation 320	0
Permutation 321	0
Permutation 322	0
Permutation 323	0
Permutation 324	0
Permutation 325	0
Permutation 326	0
Permutation 327	0
Permutation 328	0
Permutation 329	0
Permutation 330	0
Permutation 331	0
Permutation 332	584
Permutation 333	0
Permutation 334	0
Permutation 335	0
Permutation 336	0
Permutation 337	0
Permutation 338	1
Permutation 339	0
Permutation 340	0
Permutation 341	0
Permutation 342	0
Permutation 343	0
Permutation 344	0
Permutation 345	0
Permutation 346	1
Permutation 347	0
Permutation 348	0
Permutation 349	0
Permutation 350	0
Permutation 351	0
Permutation 352	1
Permutation 353	0
Permutation 354	0
Permutation 355	0
Permutation 356	0
Permutation 357	0
Permutation 358	0
Permutation 359	2
Permutation 360	0
Permutation 361	0
Permutation 362	0
Permutation 363	0
Permutation 364	0
Permutation 365	0
Permutation 366	0
Permutation 367	0
Permutation 368	0
Permutation 369	0
Permutation 370	0
Permutation 371	0
Permutation 372	0
Permutation 373	0
Permutation 374	0
Permutation 375	0
Permutation 376	0
Permutation 377	0
Permutation 378	0
Permutation 379	0
Permutation 380	0
Permutation 381	0
Permutation 382	0
Permutation 383	0
Permutation 384	0
Permutation 385	0
Permutation 386	0
Permutation 387	0
Permutation 388	0
Permutation 389	0
Permutation 390	0
Permutation 391	0
Permutation 392	0
Permutation 393	0
Permutation 394	0
Permutation 395	0
Permutation 396	0
Permutation 397	0
Permutation 398	0
Permutation 399	0
Permutation 400	0

Permutation	Number of genes with padj < 0.01
Permutation 401	0
Permutation 402	0
Permutation 403	1
Permutation 404	0
Permutation 405	0
Permutation 406	0
Permutation 407	0
Permutation 408	0
Permutation 409	0
Permutation 410	0
Permutation 411	0
Permutation 412	0
Permutation 413	0
Permutation 414	1
Permutation 415	0
Permutation 416	0
Permutation 417	0
Permutation 418	0
Permutation 419	0
Permutation 420	0
Permutation 421	0
Permutation 422	0
Permutation 423	37
Permutation 424	0
Permutation 425	0
Permutation 426	0
Permutation 427	0
Permutation 428	0
Permutation 429	8
Permutation 430	0
Permutation 431	0
Permutation 432	0
Permutation 433	0
Permutation 434	0
Permutation 435	0
Permutation 436	0
Permutation 437	0
Permutation 438	0
Permutation 439	0
Permutation 440	0
Permutation 441	0
Permutation 442	0
Permutation 443	0
Permutation 444	0
Permutation 445	0
Permutation 446	0
Permutation 447	0
Permutation 448	0
Permutation 449	0
Permutation 450	0
Permutation 451	0
Permutation 452	0
Permutation 453	0
Permutation 454	0
Permutation 455	0
Permutation 456	0
Permutation 457	1
Permutation 458	0
Permutation 459	0
Permutation 460	0
Permutation 461	0
Permutation 462	0
Permutation 463	0
Permutation 464	0
Permutation 465	0
Permutation 466	0
Permutation 467	0
Permutation 468	0
Permutation 469	0
Permutation 470	0
Permutation 471	0
Permutation 472	0
Permutation 473	0
Permutation 474	0
Permutation 475	0
Permutation 476	0
Permutation 477	0
Permutation 478	0
Permutation 479	0
Permutation 480	0
Permutation 481	0
Permutation 482	0
Permutation 483	0
Permutation 484	0
Permutation 485	0
Permutation 486	0
Permutation 487	0
Permutation 488	0
Permutation 489	0
Permutation 490	0
Permutation 491	0
Permutation 492	0
Permutation 493	0
Permutation 494	0
Permutation 495	0
Permutation 496	0
Permutation 497	0
Permutation 498	0
Permutation 499	0
Permutation 500	0

Permutation	Number of genes with padj < 0.01
**Correct Assignment**	**12**
Permutation 1	0
Permutation 2	0
Permutation 3	0
Permutation 4	0
Permutation 5	0
Permutation 6	9
Permutation 7	0
Permutation 8	0
Permutation 9	0
Permutation 10	0
Permutation 11	0
Permutation 12	0
Permutation 13	0
Permutation 14	0
Permutation 15	0
Permutation 16	0
Permutation 17	0
Permutation 18	0
Permutation 19	0
Permutation 20	0
Permutation 21	0
Permutation 22	0
Permutation 23	0
Permutation 24	0
Permutation 25	0
Permutation 26	0
Permutation 27	0
Permutation 28	0
Permutation 29	0
Permutation 30	0
Permutation 31	0
Permutation 32	0
Permutation 33	0
Permutation 34	0
Permutation 35	0
Permutation 36	0
Permutation 37	0
Permutation 38	0
Permutation 39	0
Permutation 40	0
Permutation 41	0
Permutation 42	0
Permutation 43	0
Permutation 44	0
Permutation 45	0
Permutation 46	0
Permutation 47	0
Permutation 48	0
Permutation 49	0
Permutation 50	0
Permutation 51	0
Permutation 52	0
Permutation 53	0
Permutation 54	0
Permutation 55	0
Permutation 56	0
Permutation 57	0
Permutation 58	0
Permutation 59	0
Permutation 60	0
Permutation 61	0
Permutation 62	0
Permutation 63	0
Permutation 64	0
Permutation 65	0
Permutation 66	0
Permutation 67	7
Permutation 68	2
Permutation 69	0
Permutation 70	0
Permutation 71	0
Permutation 72	0
Permutation 73	0
Permutation 74	0
Permutation 75	0
Permutation 76	0
Permutation 77	0
Permutation 78	1
Permutation 79	10
Permutation 80	0
Permutation 81	0
Permutation 82	0
Permutation 83	0
Permutation 84	1
Permutation 85	0
Permutation 86	0
Permutation 87	0
Permutation 88	0
Permutation 89	0
Permutation 90	0
Permutation 91	0
Permutation 92	0
Permutation 93	0
Permutation 94	0
Permutation 95	0
Permutation 96	0
Permutation 97	0
Permutation 98	0
Permutation 99	0
Permutation 100	0

Permutation	Number of genes with padj < 0.01
Permutation 101	0
Permutation 102	0
Permutation 103	0
Permutation 104	0
Permutation 105	0
Permutation 106	0
Permutation 107	0
Permutation 108	0
Permutation 109	0
Permutation 110	0
Permutation 111	0
Permutation 112	0
Permutation 113	0
Permutation 114	0
Permutation 115	0
Permutation 116	0
Permutation 117	0
Permutation 118	0
Permutation 119	0
Permutation 120	0
Permutation 121	0
Permutation 122	0
Permutation 123	0
Permutation 124	0
Permutation 125	1
Permutation 126	0
Permutation 127	0
Permutation 128	6
Permutation 129	0
Permutation 130	0
Permutation 131	0
Permutation 132	0
Permutation 133	0
Permutation 134	0
Permutation 135	0
Permutation 136	0
Permutation 137	0
Permutation 138	0
Permutation 139	0
Permutation 140	0
Permutation 141	0
Permutation 142	0
Permutation 143	0
Permutation 144	0
Permutation 145	0
Permutation 146	0
Permutation 147	0
Permutation 148	61
Permutation 149	0
Permutation 150	0
Permutation 151	0
Permutation 152	0
Permutation 153	0
Permutation 154	0
Permutation 155	0
Permutation 156	0
Permutation 157	0
Permutation 158	0
Permutation 159	0
Permutation 160	0
Permutation 161	0
Permutation 162	0
Permutation 163	0
Permutation 164	0
Permutation 165	1
Permutation 166	0
Permutation 167	0
Permutation 168	0
Permutation 169	0
Permutation 170	0
Permutation 171	0
Permutation 172	0
Permutation 173	0
Permutation 174	0
Permutation 175	0
Permutation 176	0
Permutation 177	0
Permutation 178	0
Permutation 179	6
Permutation 180	0
Permutation 181	0
Permutation 182	0
Permutation 183	0
Permutation 184	0
Permutation 185	0
Permutation 186	0
Permutation 187	0
Permutation 188	0
Permutation 189	0
Permutation 190	0
Permutation 191	0
Permutation 192	0
Permutation 193	0
Permutation 194	0
Permutation 195	0
Permutation 196	0
Permutation 197	0
Permutation 198	0
Permutation 199	0
Permutation 200	0

Permutation	Number of genes with padj < 0.01
Permutation 201	0
Permutation 202	0
Permutation 203	0
Permutation 204	0
Permutation 205	0
Permutation 206	0
Permutation 207	0
Permutation 208	0
Permutation 209	0
Permutation 210	0
Permutation 211	0
Permutation 212	0
Permutation 213	0
Permutation 214	0
Permutation 215	0
Permutation 216	0
Permutation 217	0
Permutation 218	0
Permutation 219	0
Permutation 220	0
Permutation 221	0
Permutation 222	0
Permutation 223	0
Permutation 224	0
Permutation 225	0
Permutation 226	0
Permutation 227	0
Permutation 228	0
Permutation 229	10
Permutation 230	0
Permutation 231	2
Permutation 232	1
Permutation 233	0
Permutation 234	0
Permutation 235	0
Permutation 236	0
Permutation 237	0
Permutation 238	0
Permutation 239	0
Permutation 240	0
Permutation 241	0
Permutation 242	0
Permutation 243	0
Permutation 244	1
Permutation 245	0
Permutation 246	0
Permutation 247	0
Permutation 248	0
Permutation 249	0
Permutation 250	0
Permutation 251	0
Permutation 252	0
Permutation 253	0
Permutation 254	0
Permutation 255	0
Permutation 256	0
Permutation 257	106
Permutation 258	0
Permutation 259	0
Permutation 260	0
Permutation 261	0
Permutation 262	0
Permutation 263	0
Permutation 264	1
Permutation 265	0
Permutation 266	0
Permutation 267	0
Permutation 268	0
Permutation 269	0
Permutation 270	0
Permutation 271	0
Permutation 272	0
Permutation 273	0
Permutation 274	0
Permutation 275	0
Permutation 276	0
Permutation 277	0
Permutation 278	0
Permutation 279	0
Permutation 280	0
Permutation 281	0
Permutation 282	0
Permutation 283	1
Permutation 284	0
Permutation 285	0
Permutation 286	0
Permutation 287	0
Permutation 288	0
Permutation 289	0
Permutation 290	0
Permutation 291	1
Permutation 292	0
Permutation 293	0
Permutation 294	8
Permutation 295	0
Permutation 296	0
Permutation 297	0
Permutation 298	0
Permutation 299	0
Permutation 300	0

Permutation	Number of genes with padj < 0.01
Permutation 301	0
Permutation 302	0
Permutation 303	0
Permutation 304	0
Permutation 305	0
Permutation 306	0
Permutation 307	0
Permutation 308	0
Permutation 309	0
Permutation 310	0
Permutation 311	1
Permutation 312	0
Permutation 313	0
Permutation 314	0
Permutation 315	0
Permutation 316	0
Permutation 317	0
Permutation 318	0
Permutation 319	0
Permutation 320	0
Permutation 321	0
Permutation 322	0
Permutation 323	0
Permutation 324	0
Permutation 325	0
Permutation 326	0
Permutation 327	1
Permutation 328	0
Permutation 329	0
Permutation 330	0
Permutation 331	0
Permutation 332	0
Permutation 333	0
Permutation 334	0
Permutation 335	0
Permutation 336	0
Permutation 337	0
Permutation 338	0
Permutation 339	0
Permutation 340	0
Permutation 341	0
Permutation 342	0
Permutation 343	0
Permutation 344	0
Permutation 345	0
Permutation 346	0
Permutation 347	0
Permutation 348	0
Permutation 349	0
Permutation 350	0
Permutation 351	0
Permutation 352	0
Permutation 353	0
Permutation 354	0
Permutation 355	0
Permutation 356	0
Permutation 357	0
Permutation 358	0
Permutation 359	0
Permutation 360	0
Permutation 361	0
Permutation 362	0
Permutation 363	0
Permutation 364	0
Permutation 365	0
Permutation 366	0
Permutation 367	0
Permutation 368	0
Permutation 369	0
Permutation 370	0
Permutation 371	0
Permutation 372	0
Permutation 373	0
Permutation 374	0
Permutation 375	0
Permutation 376	0
Permutation 377	1
Permutation 378	0
Permutation 379	0
Permutation 380	0
Permutation 381	0
Permutation 382	0
Permutation 383	0
Permutation 384	0
Permutation 385	0
Permutation 386	0
Permutation 387	0
Permutation 388	0
Permutation 389	0
Permutation 390	0
Permutation 391	0
Permutation 392	0
Permutation 393	0
Permutation 394	8
Permutation 395	0
Permutation 396	0
Permutation 397	0
Permutation 398	0
Permutation 399	0
Permutation 400	0

Permutation	Number of genes with padj < 0.01
Permutation 401	0
Permutation 402	0
Permutation 403	0
Permutation 404	0
Permutation 405	0
Permutation 406	0
Permutation 407	0
Permutation 408	0
Permutation 409	0
Permutation 410	0
Permutation 411	0
Permutation 412	0
Permutation 413	0
Permutation 414	0
Permutation 415	0
Permutation 416	0
Permutation 417	0
Permutation 418	0
Permutation 419	0
Permutation 420	0
Permutation 421	0
Permutation 422	0
Permutation 423	0
Permutation 424	1
Permutation 425	0
Permutation 426	0
Permutation 427	0
Permutation 428	0
Permutation 429	0
Permutation 430	1
Permutation 431	0
Permutation 432	0
Permutation 433	0
Permutation 434	0
Permutation 435	0
Permutation 436	0
Permutation 437	0
Permutation 438	0
Permutation 439	0
Permutation 440	0
Permutation 441	0
Permutation 442	0
Permutation 443	0
Permutation 444	0
Permutation 445	0
Permutation 446	0
Permutation 447	0
Permutation 448	0
Permutation 449	0
Permutation 450	0
Permutation 451	0
Permutation 452	0
Permutation 453	0
Permutation 454	0
Permutation 455	0
Permutation 456	0
Permutation 457	0
Permutation 458	0
Permutation 459	0
Permutation 460	0
Permutation 461	0
Permutation 462	1
Permutation 463	0
Permutation 464	0
Permutation 465	0
Permutation 466	0
Permutation 467	0
Permutation 468	0
Permutation 469	0
Permutation 470	2
Permutation 471	0
Permutation 472	0
Permutation 473	0
Permutation 474	0
Permutation 475	0
Permutation 476	0
Permutation 477	0
Permutation 478	0
Permutation 479	0
Permutation 480	0
Permutation 481	0
Permutation 482	0
Permutation 483	0
Permutation 484	0
Permutation 485	0
Permutation 486	0
Permutation 487	0
Permutation 488	0
Permutation 489	0
Permutation 490	0
Permutation 491	0
Permutation 492	0
Permutation 493	0
Permutation 494	0
Permutation 495	0
Permutation 496	0
Permutation 497	1
Permutation 498	0
Permutation 499	0
Permutation 500	0

Supplementary Table 5 | Top 10 learning vector quantization feature set (LVQ gene set) distinguished between individual cancer or precancerous condition with respect to non-cancer

Pair	Top 10 LVQ gene
HCC vs NC	FGB
HCC vs NC	FGA
HCC vs NC	CP
HCC vs NC	C3
HCC vs NC	IFITM3
HCC vs NC	FGG
HCC vs NC	ATP1B1
HCC vs NC	HRG
HCC vs NC	APOE
HCC vs NC	DHCR24
MM vs NC	NEK2
MM vs NC	CENPE
MM vs NC	HBG2
MM vs NC	NUSAP1
MM vs NC	HBG1
MM vs NC	ELL2
MM vs NC	AIDA
MM vs NC	CA1
MM vs NC	CPOX
MM vs NC	EPB42
MGUS vs NC	FPR3
MGUS vs NC	SMC4
MGUS vs NC	TRADD
MGUS vs NC	TXNDC16
MGUS vs NC	VCAM1
MGUS vs NC	ASPM
MGUS vs NC	DNAH1
MGUS vs NC	AL121603.2
MGUS vs NC	FAM109B
MGUS vs NC	FMN1
Cirr vs NC	ABCB7
Cirr vs NC	HIST1H2BF
Cirr vs NC	PSIP1
Cirr vs NC	TMEM150C
Cirr vs NC	ZC3H6
Cirr vs NC	C9orf16
Cirr vs NC	CPQ
Cirr vs NC	DYNC1I2
Cirr vs NC	ECM1
Cirr vs NC	HIST1H2AE
