# Supplementary material for: Plasma cell-free RNA profiling distinguishes cancers from pre-malignant conditions in solid and hematologic malignancies
Source: NPJ Precis Oncol. 2022 Apr 25;6:28. doi: 10.1038/s41698-022-00270-y (PMC9038724; doi:10.1038/s41698-022-00270-y)
Supplement: Supplementary file 2 — REPORTING SUMMARY [file 41698_2022_270_MOESM2_ESM.pdf]

nature research

Corresponding author(s): DBPR-NPJPRECISIONONCOLOGY-01625R

Reporting Summary

Nature Research wishes to improve the reproducibility of the work that we publish. This form provides structure for consistency and transparency in reporting. For further information on Nature Research policies, see Authors & Referees and the Editorial Policy Checklist.

Statistical parameters

When statistical analyses are reported, confirm that the following items are present in the relevant location (e.g. figure legend, table legend, main text, or Methods section).

n/a	Confirmed	
☐	☒	The exact sample size (*n*) for each experimental group/condition, given as a discrete number and unit of measurement
☐	☒	An indication of whether measurements were taken from distinct samples or whether the same sample was measured repeatedly
☐	☒	The statistical test(s) used AND whether they are one- or two-sided<br>*Only common tests should be described solely by name; describe more complex techniques in the Methods section.*
☐	☒	A description of all covariates tested
☐	☒	A description of any assumptions or corrections, such as tests of normality and adjustment for multiple comparisons
☐	☒	A full description of the statistics including central tendency (e.g. means) or other basic estimates (e.g. regression coefficient) AND variation (e.g. standard deviation) or associated estimates of uncertainty (e.g. confidence intervals)
☐	☒	For null hypothesis testing, the test statistic (e.g. *F*, *t*, *r*) with confidence intervals, effect sizes, degrees of freedom and *P* value noted<br>*Give P values as exact values whenever suitable.*
☒	☐	For Bayesian analysis, information on the choice of priors and Markov chain Monte Carlo settings
☐	☒	For hierarchical and complex designs, identification of the appropriate level for tests and full reporting of outcomes
☐	☒	Estimates of effect sizes (e.g. Cohen's *d*, Pearson's *r*), indicating how they were calculated
☐	☒	Clearly defined error bars<br>*State explicitly what error bars represent (e.g. SD, SE, CI)*

Our web collection on statistics for biologists may be useful.

Software and code

Policy information about availability of computer code

Data collection	*Provide a description of all commercial and custom code used to collect the data in this study, specifying the version used OR state that no software was used.*
Data analysis	Sequencing data processing and quality control: Each sample was sequenced to more than 20 million paired-end reads using an Illumina Nextseq or HiSeq sequencer. Adapter sequences were trimmed using sickle tool [60]. After trimming, the quality of the reads were checked using FastQC (v0.11.7) [61, 62] and RSeQC (v2.6.4) [63]. Reads were aligned to the hg38 human genome using the STAR aligner (v2.5.3a) [64] with two pass mode flag. Duplicated reads were removed using the picard tool (v1.119) [65]. Read counts for each gene were calculated using the htseq-count tool (v0.11.2) [66] in intersection-strict mode. The number of mapped reads to each gene were normalized to the total number of reads in the whole transcriptome (Reads Per Million - RPM). For each sample, we calculated exon, intron, intergenic fractions and protein coding fractions (CDS exons) using RSeQC [67] and the read_distribution.py script. Samples with an exon fraction larger than 0.35 were kept for further analysis.<br>Identification of cfRNA biomarkers (DESeq and LVQ and GO analysis):<br>Two independent methods were applied to select cfRNA features for building classification models. Differentiating genes between all pairwise comparisons were identified with the R package DESeq2 (v1.24.0) using the Wald test [68] with adjusted p-value (padj) < 0.01 (Supplemental Table S3) were used as one feature set (DE gene set). The second method for feature selection using the LVQ algorithm built in an R package caret (v6.0-84) - with 10 fold cross validation repeated 3 times [69]. The top 10 most important features were selected by ranking the varImp parameter (LVQ gene set) (Supplemental Table S5). Gene Ontology (GO) analysis was implemented on the top differentiating genes from the DESeq2 analysis with padj < 0.01 using the package topGO (v2.37.0) and a Fischer statistical test to measure significant enrichment of each Gene Ontology term [70].

Cancer type classification (LDA and RF): Two methods were used to build models for classifying cancer types using feature sets identified from pairwise comparison using DESeq2 and LVQ methods. LDA models were built using the R package MASS (v7.3-51.4) [71]. Random Forest models were built using the R package randomForest (v4.6-14) [72].

For manuscripts utilizing custom algorithms or software that are central to the research but not yet described in published literature, software must be made available to editors/reviewers upon request. We strongly encourage code deposition in a community repository (e.g. GitHub). See the Nature Research guidelines for submitting code & software for further information.

Data

Policy information about availability of data

All manuscripts must include a data availability statement. This statement should provide the following information, where applicable:

- Accession codes, unique identifiers, or web links for publicly available datasets
- A list of figures that have associated raw data
- A description of any restrictions on data availability

Data and materials availability: cfRNA sequencing data have been deposited in the Gene Expression Omnibus Repository (GSE182824).

Field-specific reporting

Please select the best fit for your research. If you are not sure, read the appropriate sections before making your selection.

☒ Life sciences ☐ Behavioural & social sciences

For a reference copy of the document with all sections, see nature.com/authors/policies/ReportingSummary-flat.pdf

Life sciences

Study design

All studies must disclose on these points even when the disclosure is negative.

Sample size	The pilot set includes 10 MM and 8 HCC patients; 12 patients with pre-malignant conditions including 9 MGUS and 4 Cirr; and 20 NC donors. The validation set includes 10 NC controls, 9 MM patients and 20 HCC patients.
Data exclusions	Sequencing data of samples with the exon fraction of less than 0.35 were excluded.
Replication	Each sample was sequenced once without replicates.
Randomization	Samples were randomly shuffled for RNA extraction, library preparation and sequencing
Blinding	Investigators were not blinded during sample collection and analysis due to the collection process from specific clinics.

Materials & experimental systems

Policy information about availability of materials

n/a	Involved in the study
☒	☐ Unique materials
☒	☐ Antibodies
☒	☐ Eukaryotic cell lines
☒	☐ Research animals
☐	☒ Human research participants

Human research participants

Policy information about studies involving human research participants

Population characteristics	Samples were collected from non-cancer subjects, and patients with diagnosis of cirrhosis, HCC, monoclonal gammopathy of undetermined significance (MGUS), and multiple myeloma. Age restricted is above 18 years old. There was no restriction on gender and race.

Method-specific reporting

n/a	Involved in the study
☒	☐ ChIP-seq
☒	☐ Flow cytometry
☒	☐ Magnetic resonance imaging
